# Genomic regions, candidate genes, and pleiotropic variants associated with physiological and anatomical indicators of heat stress response in lactating sows

**DOI:** 10.1186/s12864-024-10365-4

**Published:** 2024-05-13

**Authors:** Letícia Fernanda de Oliveira, Renata Veroneze, Katiene Régia Silva Sousa, Henrique A. Mulim, André Campelo Araujo, Yijian Huang, Jay S. Johnson, Luiz F. Brito

**Affiliations:** 1 Department of Animal Science, Federal University of Viçosa, Viçosa, MG Brazil; 2 Department of Animal Sciences, Purdue University, West Lafayette, IN USA; 3 Department of Oceanography and Limnology, Federal University of Maranhão, São Luís, MA Brazil; 4 Smithfield Premium Genetics, Rose Hill, NC USA; 5 USDA-ARS Livestock Behavior Research Unit, West Lafayette, IN USA

**Keywords:** Climatic resilience, Pleiotropy, Landrace, Large White, Maternal-line pigs, GWAS

## Abstract

**Background:**

Heat stress (HS) poses significant threats to the sustainability of livestock production. Genetically improving heat tolerance could enhance animal welfare and minimize production losses during HS events. Measuring phenotypic indicators of HS response and understanding their genetic background are crucial steps to optimize breeding schemes for improved climatic resilience. The identification of genomic regions and candidate genes influencing the traits of interest, including variants with pleiotropic effects, enables the refinement of genotyping panels used to perform genomic prediction of breeding values and contributes to unraveling the biological mechanisms influencing heat stress response. Therefore, the main objectives of this study were to identify genomic regions, candidate genes, and potential pleiotropic variants significantly associated with indicators of HS response in lactating sows using imputed whole-genome sequence (WGS) data. Phenotypic records for 18 traits and genomic information from 1,645 lactating sows were available for the study. The genotypes from the PorcineSNP50K panel containing 50,703 single nucleotide polymorphisms (SNPs) were imputed to WGS and after quality control, 1,622 animals and 7,065,922 SNPs were included in the analyses.

**Results:**

A total of 1,388 unique SNPs located on sixteen chromosomes were found to be associated with 11 traits. Twenty gene ontology terms and 11 biological pathways were shown to be associated with variability in ear skin temperature, shoulder skin temperature, rump skin temperature, tail skin temperature, respiration rate, panting score, vaginal temperature automatically measured every 10 min, vaginal temperature measured at 0800 h, hair density score, body condition score, and ear area. Seven, five, six, two, seven, 15, and 14 genes with potential pleiotropic effects were identified for indicators of skin temperature, vaginal temperature, animal temperature, respiration rate, thermoregulatory traits, anatomical traits, and all traits, respectively.

**Conclusions:**

Physiological and anatomical indicators of HS response in lactating sows are heritable but highly polygenic. The candidate genes found are associated with important gene ontology terms and biological pathways related to heat shock protein activities, immune response, and cellular oxidative stress. Many of the candidate genes with pleiotropic effects are involved in catalytic activities to reduce cell damage from oxidative stress and cellular mechanisms related to immune response.

**Supplementary Information:**

The online version contains supplementary material available at 10.1186/s12864-024-10365-4.

## Background

Global warming has become a major concern for the long-term sustainability of livestock production. The ten warmest years on record since 1880 have all occurred after 2010, and the nine years from 2014 to 2022 are the nine warmest years on record [1]. These higher temperatures have a direct impact on animal production systems due to increased incidence of heat stress (HS) [2]. The absence of functional sweat glands in pigs limits their ability to use evaporative heat loss through sweating, reducing their thermoregulation ability in hot environments [2, 3]. Furthermore, intensive genetic selection for greater productive and reproductive performance has been associated with an increased metabolic heat production, making the animals more sensitive to high environmental temperatures [4]. In this context, breeding for greater heat tolerance could improve the animals’ ability to cope with HS conditions.

Breeding pigs for improved resilience to HS is possible as there is genetic variability in their response to HS [5–8]. One alternative for genetically identifying animals that perform better under HS conditions is through the use of reaction norm approach that model the genetic merit of an individual over an environmental gradient [9]. In general, reaction norm models are based on variability in performance traits across a continuous environmental gradient. Estimated breeding values for heat tolerance (slope of the reaction norms) based on this approach tend to be unfavorably correlated with the level of production (e.g [7]). Therefore, identifying physiological, behavioral, and anatomical indicators of HS response that could be incorporated in selection indexes to breed for improved climatic resilience is promising in modern pig breeding programs [10]. Breeding for indicators of HS response could capture additional biological mechanisms involved in HS response and enable improvements in climatic resilience without compromising productivity [11]. Johnson et al. [10] described several traits as HS response indicators, such as skin and automatically-recorded vaginal temperatures, and respiration rate, and Freitas et al. [11] showed that these traits are heritable, i.e., they can be improved through genetic selection. Understanding the genetic background of these novel traits is a key step before including them in a breeding scheme. This includes the detection of quantitative trait loci (QTL) and the identification of candidate genes and biological pathways controlling the phenotypic expression of these HS indicator traits.

Genome-wide association studies (GWAS) enable the identification of genomic markers statistically associated with the traits of interest. GWAS is based on the principle of identifying genomic markers in linkage disequilibrium (LD) with the QTL affecting the trait of interest [12]. Various studies have reported genomic regions associated with heat tolerance based on reaction norm models for reproductive traits [6] and productive traits such as hot carcass weight [8, 13]. Kim et al. [14] did not identify significant markers associated with respiration rate, rectal temperature, and skin temperature in crossbred gilts, however, they did report some genes known to be involved in physiological adaptation to general stressors. However, there is still a lack of studies aiming to identify candidate genes associated with physiological indicators of HS, such as vaginal temperature, skin temperature, and respiration rate in pigs using many phenotypic records and genomic markers.

Heat stress indicator traits may share metabolic pathways. A single locus can be involved in the expression of more than one trait, which is a phenomenon known as pleiotropy [15]. Pleiotropy occurs when a single causal variant or multiple variants within the same gene (or region) are associated with different phenotypes. Furthermore, pleiotropy also occurs when one phenotype causes a second phenotype, and a genetic variant is directly associated with the initial phenotype [16]. Pleiotropy is one of the causes of genetic correlation between traits. In this context, Freitas et al. [11] reported positive and moderate to high genetic correlations among physiological indicators of HS response such as skin temperature, vaginal temperature, panting score, and respiration rate. Identifying genes with pleiotropic effects and the genetic correlation between traits allows breeders to understand the biological results of the correlated response in multiple-trait selection. In this context, the primary objectives of this study were to identify genomic regions associated with physiological and anatomical indicators of HS response in lactating sows using imputed whole-genome sequence (WGS) data and identify pleiotropic variants. In addition, candidate genes, gene ontology (GO) terms, and metabolic pathways were identified in the significant genomic regions associated with the HS responses of lactating sows.

## Results

### Overview

We performed GWAS for 18 indicators of HS response and anatomical traits in crossbred lactating sows (Large White x Landrace) and found candidate genes and pleiotropic variants affecting these traits. The thermoregulatory indicator traits evaluated were ear skin temperature (T_ES_), shoulder skin temperature (T_SS_), rump skin temperature (T_RS_), tail skin temperature (T_TS_), respiration rate (RR), panting score (PS), vaginal temperature automatically measured every 10 min (T_Vall_), the average of the six records per hour corresponding to 0800, 1200, 1600, and 2000 h during four days (T_V4days_), and single records corresponding to 0800, 1200, 1600, and 2000 h measured at the first day of collection (T_V8h_, T_V12h_, T_V16h_, T_V20h_, respectively). The anatomical traits evaluated were hair density (HD), body size (BS), body condition score (BCS) using a sow caliper (BCS_cal_), visual BCS (BCS_vis_), ear length (EL), and ear area (EA). The variance components and genetic parameters for the traits evaluated in this study have been previously reported by Freitas et al. [11]. A description of all the studied traits and their corresponding heritability estimates, as previously reported by Freitas et al. [11], is presented in Table 1. The significant SNPs were identified based on their respective *p*-values, with adjustment for multiple testing using the Bonferroni correction at a significance level of α = 0.05 for genome-wide significance. To take into account the dependence among tests due to linkage disequilibrium, α was divided by the number of independent chromosome segments ($Me$).

**Table 1:** Description of the evaluated traits and heritability estimates reported by Freitas et al. [11]

Trait Name	Trait abbreviation	^a^Description		^b^h^2^
Ear skin temperature	T_ES_	Ear skin surface temperature collected at 0800, 1200, 1600, and 2000 h daily during a period of four consecutive days.		0.04 ± 0.01
Shoulder skin temperature	T_SS_	Shoulder skin surface temperature collected at 0800, 1200, 1600, and 2000 h daily during a period of four consecutive days.		0.06 ± 0.01
Rump skin temperature	T_RS_	Rump skin surface temperature collected at 0800, 1200, 1600, and 2000 h daily during a period of four consecutive days.		0.06 ± 0.01
Tail skin temperature	T_TS_	Tail skin surface temperature collected at 0800, 1200, 1600, and 2000 h daily during a period of four consecutive days.		0.05 ± 0.01
Respiration Rate	RR	Respiration rate was measured by counting flank movements for 15 s and multiplying by 4 to calculate breaths per minute (bpm) at 0800, 1200, 1600, and 2000 h daily during four consecutive days.		0.06 ± 0.01
Panting score	PS	A subjective panting score assessed at 15:30 h during four consecutive days and scored from 0 to 3 (0: if it was with closed mouth and normal breathing; 1: closed mouth and rapid breathing; 2: open mouth and rapid breathing; 3: open mouth and rapid breathing with obvious salivation).		0.05 ± 0.01
Vaginal temperature - All records	T_Vall_	Vaginal temperature automatically recorded in 10 min intervals over four days using calibrated thermochron temperature recorders.		0.15 ± 0.02
Vaginal temperature − 4 days	T_V4days_	The average vaginal temperature of the six records per hour corresponding to 0800, 1200, 1600, and 2000 h during four consecutive days.		0.22 ± 0.03
Vaginal temperature at 8:00 h	T_V8h_	The average vaginal temperature of the six records per hour corresponding to 0800 h on the first day of collection.		0.25 ± 0.05
Vaginal temperature at 12:00 h	T_V12h_	The average vaginal temperature of the six records per hour corresponding to 1200 h on the first day of collection.		0.29 ± 0.05
Vaginal temperature at 16:00 h	T_V16h_	The average vaginal temperature of the six records per hour corresponding to 1600 h on the first day of collection.		0.22 ± 0.03
Vaginal temperature at 20:00 h	T_V20h_	The average vaginal temperature of the six records per hour corresponding to 2000 h on the first day of collection.		0.22 ± 0.03
Hair density	HD	A subjective visual score ranging from 0 to 2, being the score 0 = hairless or limited hair cover, 1 = normal or moderate hair cover, and 2 = sow with greater than normal hair cover.		0.57 ± 0.09
Body size	BS	A visual score of the animal’s body size into three categories: small, medium, or large.		0.53 ± 0.06
Body condition score - caliper	BCS_cal_	Body condition score measured using a sow caliper tool.		0.60 ± 0.07
Body condition score - visual	BCS_vis_	A visual body condition score based on five categories: 1 = emaciated, 2 = thin, 3 = ideal, 4 = fat, and 5 = overly fat.		0.53 ± 0.07
Ear area	EA	The ear area was calculated using a photo taken with a digital camera, where a 10.2 × 15.2 cm grid card containing 1 cm × 1 cm squares was placed next to the sows’ ear.		0.57 ± 0.12
Ear length	EL	The ear length was calculated using a photo taken with a digital camera, where a 10.2 × 15.2 cm grid card containing 1 cm × 1 cm squares was placed next to the sows’ ear.		0.53 ± 0.12

^a^Data collection protocols have been described by Johnson et al. [10] and Freitas et al. [11]

^b^These heritability estimates were previously reported by Freitas et al. [11]

### Genome-wide association and post-GWAS indicators of heat stress response in lactating sows

**Fig. 1:**
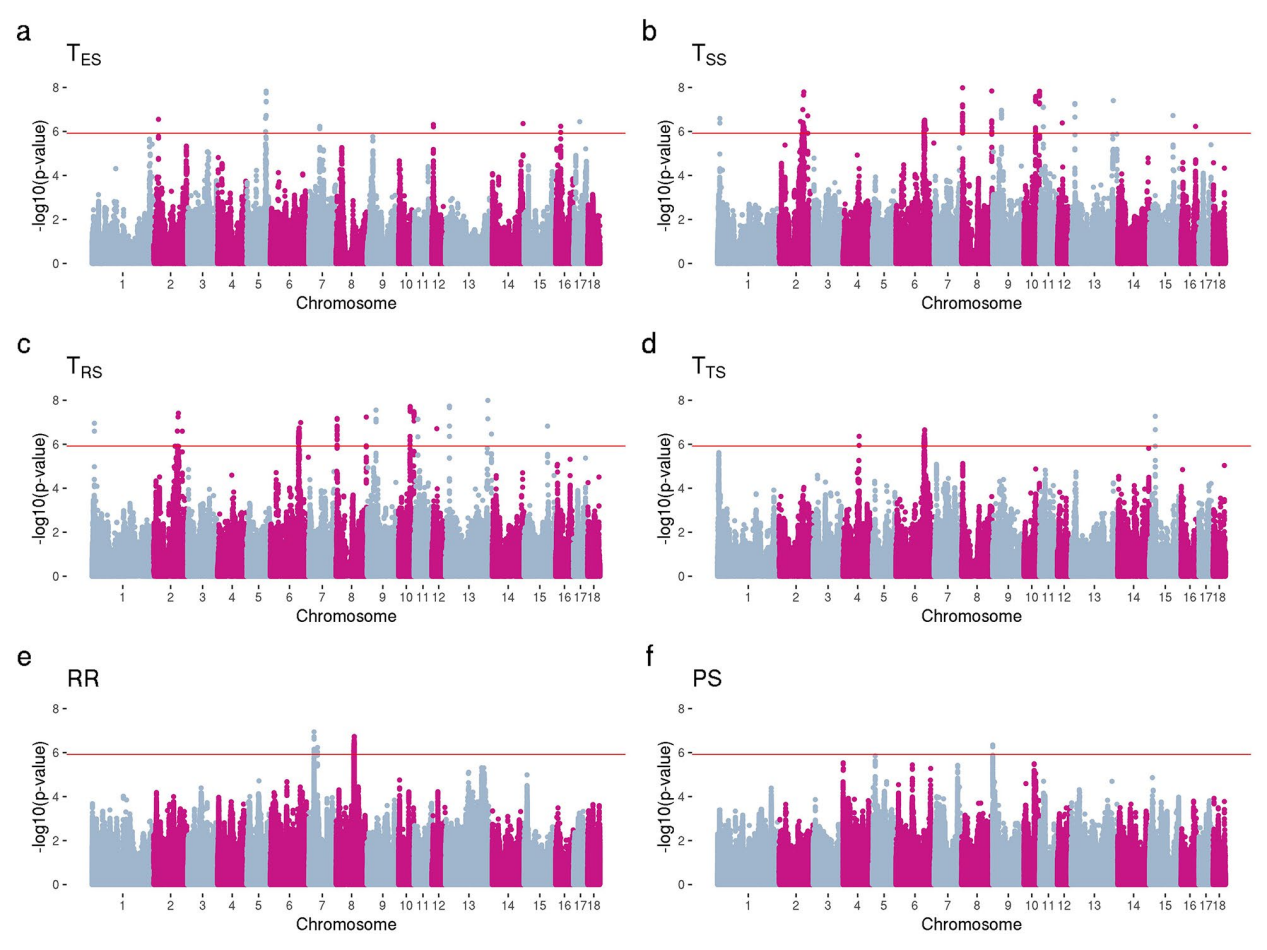
Manhattan plots for skin temperatures, respiration rate, and panting score using whole-genome sequence data. -log10(*p*-values) for ear skin temperature (T_ES_; **a**), shoulder skin temperature (T_SS_; **b**), rump skin temperature (T_RS_; **c**), tail skin temperature (T_TS_; **d**), respiration rate (RR; **e**), and panting score (PS; **f**). Genome-wide significance level is shown in a red line

The medium-density genotypes were imputed to WGS using the Swine Imputation (SWIM) Server [17] and the imputed genotypes were used to perform GWAS for all traits included in the study. A total of 1,388 SNPs across 16 chromosomes were associated with T_ES_, T_SS_, T_RS_, T_TS_, RR, PS, T_Vall_, T_V8h_, HD, BCS_cal_, and EA (Table S1). T_SS_ and T_RS_ are influenced by many significant genomic regions located on many chromosomes (Fig. 1.b-c), indicating that these traits are highly polygenic. For T_ES_, significant peaks located on chromosomes 5, 7, 12, and 16 were identified (Fig. 1.a), while for T_TS_, we observed significant peaks located on chromosomes 4, 6, and 15 (Fig. 1.d). Two significant genomic regions located on chromosomes 7 and 8 were identified for RR (Fig. 1.e) and one significant region on chromosome 9 was found to be associated with PS (Fig. 1.f).

**Fig. 2:**
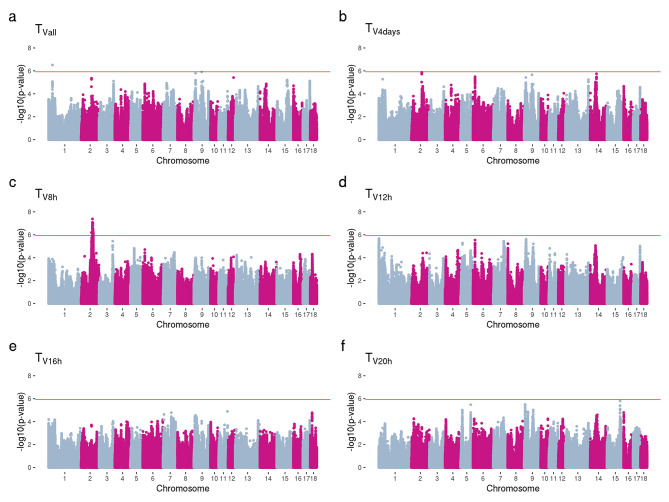
Manhattan plots of GWAS for vaginal temperatures using whole-genome sequence data. -log10(*p*-values) for all measures (every 10 min) of vaginal temperatures for four days (TV_all_; **a**), four-time measures of vaginal temperatures for four days (T_V4days_; **b**), vaginal temperature measured on the first day at 8:00 (T_V8h_; **c**), at 12:00 (T_V12h_; **d**), at 16:00 (T_V16h_; **e**), and at 20:00 (T_V20h_; **f**). Genome-wide significance level is shown in a red line

**Fig. 3:**
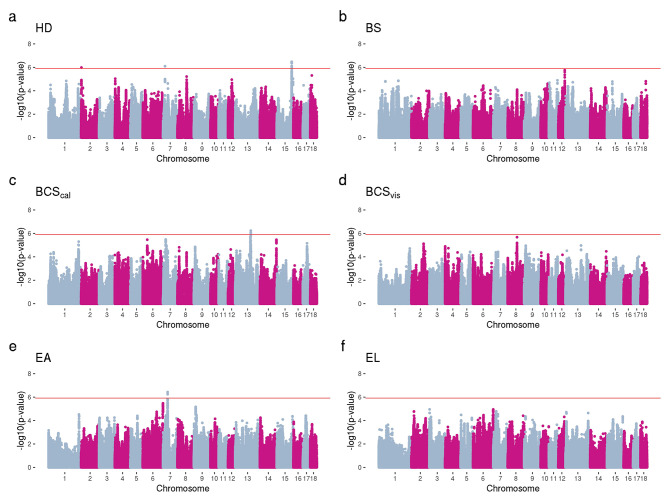
Manhattan plots of GWAS for anatomical traits using whole-genome sequence data. -log10(*p*-values) for hair density (HD; **a**), body size (BS; **b**), body condition score using a sow caliper (BCS_cal_; **c**) and visual (BCS_vis_; **d**), ear area (EA; **e**), and ear length (EL; **f**). Genome-wide significance level shown in a red line

The Manhattan plots for vaginal temperature traits are shown in Fig. 2. One significant marker was observed on chromosome 1 (Fig. 2.a) for T_Vall,_ and one significant peak (228 SNPs) on chromosome 2 for T_V8h_ (Fig. 2.c). No significant SNPs were observed for T_V4days_, T_V12h_, T_V16h_, and T_V20h_ after Bonferroni correction for multiple tests (Fig. 2). Figure 3 shows the results of GWAS for anatomical traits. Two significant SNPs located on chromosomes 2 and 7 and a significant peak (13 SNPs) on chromosome 15 were identified for HD (Fig. 3.a). Four significant SNPs located on chromosome 13 were found to be associated with BCS_cal_ (Fig. 3.c), while three significant SNPs on chromosome 7 were identified to be associated with EA (Fig. 3.e). No significant SNPs were observed for BS, BCS_vis_, and EL after multiple testing correction (Fig. 3). The Q-Q plots for the GWAS analyses for all evaluated traits are presented in Figures S1-S3 (Additional File 1). The lambda values (genomic inflation factor) ranged from 1.00 to 1.32.

Genomic windows of 100 kb on each side of significant genomic markers were used to identify candidate genes associated with each trait, as shown in Table S2. A total of 26, 151, 102, 35, 63, eight, two, 93, four, four, and 11 genes were identified to be associated with T_ES_, T_SS_, T_RS_, T_TS_, RR, PS, T_Vall_, T_V8h_, HD, BCS_cal_, and EA, respectively. The functional enrichment analyses enabled the identification of 20 GO terms (five biological processes, four cellular components, and 11 molecular functions), and 11 metabolic pathways (Table 2). The complete list of all GO and metabolic pathways identified, including those considered to be statistically significant and suggestive, are shown in Table S3.

**Table 2:** Significant gene ontology terms and pathways for heat stress related traits

Trait	Category	Term	*p*-value	Genes
EA	Pathway	ssc04659 Th17 cell differentiation	0.0477	*HSP90AB1, NFKBIE*
RR	Biological process	GO:0042026 protein refolding	0.0322	*ENSSSCG00000029160, HSPA1L*
RR	Biological process	GO:0034620 cellular response to unfolded protein	0.0390	*ENSSSCG00000029160, HSPA1L*
RR	Biological process	GO:0007130 synaptonemal complex assembly	0.0412	*BAG6, EHMT2*
RR	Biological process	GO:0006402 mRNA catabolic process	0.0412	*LSM2, DXO*
RR	Molecular function	GO:0051787 misfolded protein binding	0.0010	*BAG6, ENSSSCG00000029160, HSPA1L*
RR	Molecular function	GO:0016597 amino acid binding	0.0171	*AARS2, DDAH2*
RR	Molecular function	GO:0002161 aminoacyl-tRNA editing activity	0.0214	*AARS2, VARS1*
RR	Molecular function	GO:0005496 steroid binding	0.0298	*CYP21A2, NR3C2*
RR	Pathway	ssc04610 Complement and coagulation cascades	0.0130	*CFB, C4A, C2*
RR	Pathway	ssc05150 Staphylococcus aureus infection	0.0169	*CFB, C4A, C2*
RR	Pathway	ssc03040 Spliceosome	0.0295	*LSM2, HSPA1L, CDC5L*
T_ES_	Molecular function	GO:0005229 intracellular calcium activated chloride channel activity	0.0182	*ANO4, TTYH2*
T_ES_	Molecular function	GO:0003777 microtubule motor activity	0.0444	*KIF19, DNAI2*
T_ES_	Molecular function	GO:0005254 chloride channel activity	0.0454	*ANO4, TTYH2*
T_RS_, T_SS_	Cellular component	GO:0098797 plasma membrane protein complex	0.0308	*EVC2, EVC*
T_RS_, T_SS_	Cellular component	GO:0005634 nucleus	0.0387	*NR1D1, THRA, ADNP2, NIF3L1, PIP4K2A, MRPS18C, ORC2, EBF1, NUDT12, CLK1, CDC6, SAMD13, PARD6G, EVC2, RNASEH2B, HSBP1L1, ABRAXAS1, HELQ, FUBP1*
T_RS_, T_SS_	Molecular function	GO:0070181 small ribosomal subunit rRNA binding	0.0271	*MRPS18C, ABRAXAS1*
T_RS_, T_SS_	Molecular function	GO:0003688 DNA replication origin binding	0.0437	*ORC2, CDC6*
T_RS_	Pathway	ssc01100 Metabolic pathways	0.0486	*STT3B, COQ2, UOX, ALG9, NUDT12, ENSSSCG00000016093, GPAT3, ENSSSCG00000003754, ENSSSCG00000024765, PIP4K2A, HPSE*
T_TS_	Biological process	GO:0007098 centrosome cycle	0.0275	*SSX2IP, PARD6G*

### Pleiotropic effects

As traits are correlated and pleiotropic effects are expected, a multiple-trait analysis, as proposed by Bolormaa et al. [18] was also performed. First, the traits were decorrelated using Cholesky transformation [19, 20] and grouped into seven categories: skin surface temperature traits (T_ES_, T_SS_, T_RS_, and T_TS_), vaginal temperatures (T_Vall_, T_V4days_, T_V8h_, T_V12h_, T_V16h_, and T_V20h_), indicators of animal temperature (i.e., all skin surface and vaginal temperature traits), respiration rate traits (RR and PS), thermoregulatory indicators (i.e., all skin surface temperatures, vaginal temperatures, and respiration traits), anatomical traits (BCS_cal_, BCS_vis_, HD, BS, EA, and EL), and a combination of all traits.

**Fig. 4:**
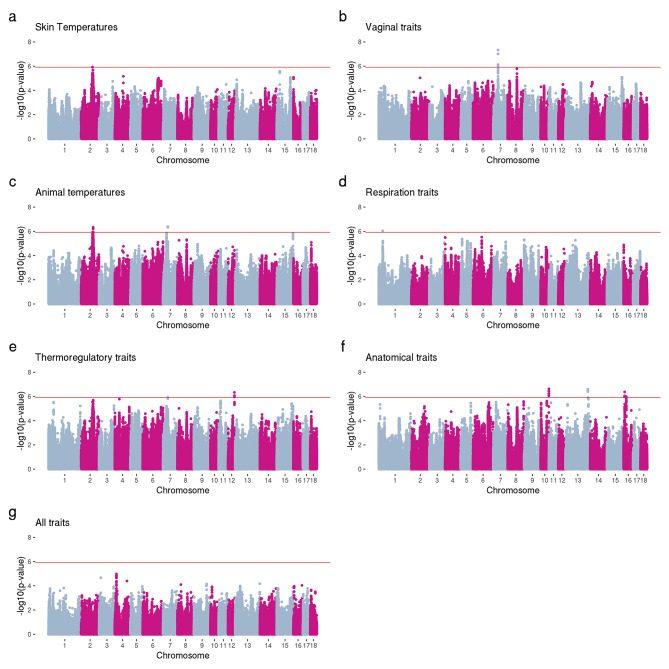
Manhattan plots for multiple-trait analysis for different categories of traits. (**a**) The category skin temperatures included the traits ear skin temperature (T_ES_), shoulder skin temperature (T_SS_), rump skin temperature (T_RS_), tail skin temperature (T_TS_). (**b**) The category vaginal temperatures included all measures (every 10 min) of vaginal temperatures for four days (TV_all_), four-time measures of vaginal temperatures for four days (T_V4days_), vaginal temperature measured on the first day at 8:00 (T_V8h_), at 12:00 (T_V12h_), at 16:00 (T_V16h_), and at 20:00 (T_V20h_). (**c**) The category animal temperature included the traits T_ES_, T_SS_, T_RS_, T_TS_, T_Vall_, T_V4days_, T_V8h_, T_V12h_, T_V16h_, and T_20h_. (**d**) The category respiration traits included respiration rate (RR) and panting score (PS). (**e**) The category thermoregulatory traits included the traits T_ES_, T_SS_, T_RS_, T_TS_, T_Vall_, T_V4days_, T_V8h_, T_V12h_, T_V16h_, T_20h_, RR, and PS. (**f**) The category anatomical traits included the traits hair density (HD), body size (BS), body condition score using a sow caliper (BCS_cal_) and visual (BCS_vis_), ear area (EA), and ear length (EL). (**g**) The category all traits included the traits (T_ES_, T_SS_, T_RS_, T_TS_, T_Vall_, T_V4days_, T_V8h_, T_V12h_, T_V16h_, T_20h_, RR, PS, HD, BS, BCS_cal_, BCS_vis_, EA, and EL). Genome-wide significance level shown in a red line

We identified one significant SNP with potential pleotropic effect for the skin temperature group of traits, six significant variants for the vaginal temperatures group of traits, six significant markers for the group of traits related to animal temperature, one significant marker for respiration rate traits, four significant markers for the thermoregulatory traits, and 21 significant markers for anatomical traits (Fig. 4). The significant marker observed for the skin temperatures group was also observed for the group containing all traits. The same significant region on chromosome 7 was observed for vaginal temperature and animal temperature groups, which was also observed for the thermoregulatory traits group.

Seven, five, six, two, seven, 15, and 14 genes were identified for the trait groups of vaginal temperature, animal temperatures, respiration traits, thermoregulatory traits, anatomical traits, and all traits, respectively (Table 3). Five common genes (*ENPP5*, *ENPP4*, *RCAN2*, *U6*, and *CLIC5*) were annotated for the vaginal temperatures, animal body temperatures, and thermoregulatory traits groups. Only one significant (*p* = 0.0269) GO term of biological process (GO:0007605 - Sensory perception of sound) for the animal temperatures group, involving the annotated genes *ADGRV1* and *CLIC5* was identified.

**Table 3:** Candidate genes with pleiotropic effects for physiological and anatomical indicators of heat stress response

Category^1^	Chr^2^	Position (bp)^3^	Genes	Biotype
Skin temperatures	2	95,549,933	*TMEM161B*	protein coding
	2	95,549,933	*ENSSSCG00000054377*	lncRNA
	2	95,549,933	*ENSSSCG00000044415*	lncRNA
	2	95,549,933	*ENSSSCG00000061590*	lncRNA
	2	95,549,933	*ENSSSCG00000053188*	lncRNA
	2	95,549,933	*ENSSSCG00000051977*	lncRNA
	2	95,549,933	*U6*	snRNA
Vaginal temperatures	7	40,890,048	*ENPP4*	protein coding
	7	40,890,048	*ENPP5*	protein coding
	7	40,890,048	*RCAN2*	protein coding
	7	40,722,404–40,890,048	*CLIC5*	protein coding
	7	40,722,404–40,890,048	*U6*	snRNA
Animal temperatures	2	98,311,250	*ADGRV1*	protein coding
	2	98,311,250	*ENSSSCG00000058343*	lncRNA
	2	98,311,250	*ENSSSCG00000060683*	lncRNA
	2	98,311,250	*ENSSSCG00000057324*	lncRNA
	2	98,311,250	*ENSSSCG00000060037*	lncRNA
	2	98,311,250	*U6*	snRNA
	2	101,726,071–101,807,832	*MCTP1*	protein coding
	2	101,807,832	*ENSSSCG00000045627*	protein coding
	2	102,512,272	*ENSSSCG00000039731*	protein coding
	2	102,512,272	*ELL2*	protein coding
	2	102,512,272	*ENSSSCG00000048636*	lncRNA
	7	40,890,048	*ENPP4*	protein coding
	7	40,890,048	*ENPP5*	protein coding
	7	40,890,048	*RCAN2*	protein coding
	7	40,722,404 − 40,890,048	*CLIC5*	protein coding
	7	40,722,404 − 40,890,048	*U6*	snRNA
Respiration traits	1	32,189,900	*AKAP7*	protein coding
	1	32,189,900	*ENSSSCG00000062940*	lncRNA
Thermoregulatory traits	7	40,890,048	*ENPP4*	protein coding
	7	40,890,048	*ENPP5*	protein coding
	7	40,890,048	*RCAN2*	protein coding
	7	40,890,048	*CLIC5*	protein coding
	7	40,890,048	*U6*	snRNA
	12	54,342,040–54,347,768	*NTN1*	protein coding
	12	54,342,040–54,347,769	*STX8*	protein coding
Anatomical traits	10	68,752,585	*ENSSSCG00000011162*	protein coding
	10	68,752,585–69,231,514	*DIP2C*	protein coding
	10	69,069,560–69,352,432	*ZMYND11*	protein coding
	10	68,752,585	*ENSSSCG00000052560*	processed pseudogene
	10	68,752,585	*ENSSSCG00000042778*	lncRNA
	10	68,752,585	*ENSSSCG00000056287*	lncRNA
	16	22,842,569	*GDNF*	protein coding
	16	22,823,916–22,842,569	*WDR70*	protein coding
	16	6,726,539	*ENSSSCG00000016796*	protein coding
	16	8,253,548	*CDH18*	protein coding
	16	8,687,034	*ENSSSCG00000050005*	protein coding
	16	6,726,539	*ENSSSCG00000051524*	lncRNA
	16	8,687,034	*ENSSSCG00000060866*	lncRNA
	16	8,687,034	*ENSSSCG00000061416*	lncRNA
	16	8,687,034	*ENSSSCG00000057598*	lncRNA
All traits	2	95,953,423	*ENSSSCG00000060814*	lncRNA
	2	95,953,423	*ENSSSCG00000063280*	lncRNA
	2	95,953,423	*ENSSSCG00000055379*	lncRNA
	2	95,953,423	*ENSSSCG00000060171*	lncRNA
	2	95,953,423	*ENSSSCG00000059644*	lncRNA
	2	95,953,423	*ENSSSCG00000056869*	lncRNA
	2	95,953,423	*ENSSSCG00000056397*	lncRNA
	2	95,953,423	*ENSSSCG00000053066*	lncRNA
	4	121,737,114	*ENSSSCG00000059304*	lncRNA
	4	121,737,114	*ENSSSCG00000057332*	lncRNA
	4	121,737,114	*ENSSSCG00000061167*	lncRNA
	7	85,032,675	*MCTP2*	protein coding
	7	85,032,675	*ENSSSCG00000035989*	protein coding
	7	85,032,675	*ENSSSCG00000054967*	lncRNA

^1^The category skin temperatures included the traits ear skin temperature (T_ES_), shoulder skin temperature (T_SS_), rump skin temperature (T_RS_), tail skin temperature (T_TS_). The category vaginal temperatures included all measures (every 10 min) of vaginal temperatures for four days (TV_all_), four-time measures of vaginal temperatures for four days (T_V4days_), vaginal temperature measured on the first day at 8:00 (T_V8h_), at 12:00 (T_V12h_), at 16:00 (T_V16h_), and at 20:00 (T_V20h_). The category animal temperature included the traits T_ES_, T_SS_, T_RS_, T_TS_, T_Vall_, T_V4days_, T_V8h_, T_V12h_, T_V16h_, and T_20h_. The category respiration traits included respiration rate (RR) and panting score (PS). The category thermoregulatory traits included the traits T_ES_, T_SS_, T_RS_, T_TS_, T_Vall_, T_V4days_, T_V8h_, T_V12h_, T_V16h_, T_20h_, RR, and PS. The category anatomical traits included the traits hair density (HD), body size (BS), body condition score using a sow caliper (BCS_cal_) and visual (BCS_vis_), ear area (EA), and ear length (EL). The category all traits included the traits (T_ES_, T_SS_, T_RS_, T_TS_, T_Vall_, T_V4days_, T_V8h_, T_V12h_, T_V16h_, T_20h_, RR, PS, HD, BS, BCS_cal_, BCS_vis_, EA, and EL)

^2^Chr: Chromosome

^3^bp: base pair

## Discussion

### Genome-wide association for heat stress indicators

The traits evaluated in this study include physiological and anatomical indicators of heat stress response in lactating sows. The physiological indicators are response to mechanisms used by the animal to dissipate heat, such as increasing body temperature and respiration rate [21, 22]. The anatomical indicators are anatomical characteristics that affect the ability of the animal to dissipate heat, such as the animal’s surface area, hair density, and body mass [23]. The heritability estimates for the traits evaluated in this study were previously reported by Freitas et al. [11]. Skin temperatures (i.e., T_ES_, T_SS_, T_RS_, and T_TS_), RR, and PS exhibited low heritability estimates, ranging from 0.04 to 0.06; vaginal temperatures (i.e., T_Vall_, T_V4days_, T_V8h_ T_V12h_, T_V16h_, and T_V20h_) were moderately heritable, with estimates ranging from 0.15 to 0.29 [11]; and, anatomical traits (i.e., BCS_cal_, BCS_vis_, HD, BS, EA, and EL) showed moderate to high heritability estimates, ranging from 0.25 to 0.40 [11].

The statistical power of GWAS is impacted by various factors, including trait heritability [24, 25]. A higher heritability increases statistical power, as heritability is a key determinant of effect size [24]. Consequently, as heritability increases, the effect size of each causal variant can also increase, thereby increasing the probability of identifying causal variants [24, 25]. Additionally, the performance of GWAS can be improved by using higher density genotyping. Several studies have shown that performing GWAS using whole-genome sequence (WGS) data can improve QTL detection [26–28] because it contains most causal mutations in the genome and can enable the identification of variants that are highly linked and closer to the causal mutations as compared to genotyping platforms with less SNPs. However, sequencing a large number of animals is still expensive. A more cost-effective approach is the use of genotype imputation from low-density to WGS data [28, 29].

In this study, the genomic inflation factor across the 18 evaluated traits ranged from 1.00 to 1.32, which suggested that the results might be slightly inflated for some traits. Although the inflation factor may indicate small population structure effects, Yang et al. [30] showed that significant inflation of test statistics is expected under polygenic inheritance even when there is no population stratification. Additionally, the genomic inflation factor reflects the trait heritability and the number of causal variants [30]. The range of observed values are within those observed in the literature for other complex traits. Multiple markers located on sixteen chromosomes, each with relatively small effect sizes (Table S1), were associated with the physiological and anatomical indicators of HS response in lactating sows, suggesting that these traits are highly polygenic. For some of the evaluated traits, no significant markers were identified, which could be due to the study sample size or the polygenic nature of the study traits. Polygenic traits are influenced by numerous loci with small effects on trait expression, reducing the likelihood of identifying significant markers in association studies [31]. Furthermore, identifying markers associated with such traits requires larger sample sizes and more phenotypic records to enhance the power of the association analyses [31]. The amount of data used in this study might not be sufficient to identify associated markers with some of these traits (e.g., T_V4days_, T_V12h_).

Some genomic regions were commonly found to be significant for the skin surface temperature traits, which was already expected since these traits are highly genetically correlated (from 0.78 to 0.91) [11]. Additionally, Johnson et al. [10] reported moderate to high phenotypic correlations for these same traits (from 0.56 to 0.76). As these traits are highly genetically correlated, genetically selecting for skin temperature measured at any of the locations evaluated in this study should result in similar genetic progress on all of them due to indirect genetic responses.

A significant genomic region located on chromosome 2 was associated with vaginal temperature measured at 800 h. The genes harboring this region included *RASA1* (Ras GTPase-Activating Protein 1), a regulator of blood vessel and lymphatic vessel growth in adult mice and humans. *RASA1* induced-deficiency results in a lymphatic vessel disorder characterized by hyperplasia and leakage [32], which can affect the ability to vasodilate and dissipate excess body heat through the skin. Another gene found in the significant genomic region for T_V8h_ was *LYSMD3* (LysM Domain Containing 3). This gene plays a crucial role in the innate immune response by recognizing chitin and fungal spores, mediating the production of cytokines, and promoting early inflammatory responses in human lung epithelial cells [33]. For T_Vall_, the candidate gene *AKAP7* (A-kinase Anchoring Protein 7), which encodes a protein kinase A-binding scaffolding molecule, was identified. This gene is crucial in cellular regulatory pathways during viral infections and influences neuroprotection by enhancing mitochondrial networks and inhibiting apoptosis under glutamate-induced oxidative stress, being reported as a cytoprotective factor, potentially preventing tissue damage during viral infections [34–36]. Due to *AKAP7*’s cytoprotective functions, it might also contribute to preventing tissue damage in HS conditions. Investigating the same population as in this study, Wen et al. [37] used random regression models to analyze all automatically-recorded vaginal temperature records as longitudinal traits. Wen et al. [37] identified two important genomic regions on chromosomes 10 and 16, containing genes associated with immunity, protein transport, and energy metabolism. However, in this study, we did not find these same genomic regions and genes, which might be due to different modeling approaches and the small effect size of the identified genomic regions.

It is well-known that heat shock proteins (HSP) play an important role in cell survival under stress conditions, especially HS [38–40]. HSP protects cells from damage by binding to unfolded or denatured proteins, preventing their aggregation and promoting their refolding [41, 42]. In addition, HSP helps to restore protein structure and function, ensuring that essential processes can continue despite the stress [41, 42]. HSP also acts in the degradation of proteins that cannot be refolded properly and can interact with other proteins and modulate their activity or stability, influencing various cellular processes such as cell cycle progression and apoptosis [41]. HSP activities have been reported to minimize HS-induced cellular death and improve thermotolerance in livestock [43] and to regulate the immune response, including stimulatory and regulatory functions [44]. In this study, we found genomic regions associated with T_SS_, T_RS_, and T_TS_ overlapping with *HSBP1L1* (Heat Shock Factor Binding Protein 1 Like 1) and regions associated with EA overlapping with the *HSP90AB1* (Heat Shock Protein 90 Alpha Family Class B Member 1) gene. *HSBP1L1* is presumed to suppress the heat shock factor transcription under stress, suggesting that *HSBP1L1* may regulate cellular responses to stress and cellular heat acclimation [45]. *HSP90AB1* encodes a member of the heat shock protein 90 family, a crucial protein for signal transduction, protein folding, and degradation, playing a significant role in maintaining protein stability and promoting cell survival, particularly under cellular stress [46]. Genetic variations in *HSP90AB1* have been linked to stress-induced mortality, heat tolerance, and productivity in animals [46].

One of the main responses to HS and mechanisms used for pigs to maintain their body temperature is increased RR, allowing them to dissipate heat through evaporation [4, 47, 48]. In the genomic windows around the two significant regions associated with RR we identified 63 genes, including *HSPA1L* (Heat Shock Protein Family A -Hsp70 - Member 1 Like), which encodes a 70 kDa heat shock protein crucial for cellular and molecular responses to HS in livestock. *HSPA1L* ensures proper protein folding, unfolding, and refolding, having protective functions alongside other HSPs during HS events [49]. In the functional analyses, which includes all identified candidate genes for RR, a biological process related to protein refolding and cellular response to unfolded protein was revealed based on the GO analyses. Molecular functions of misfolded protein binding and amino acid binding were also identified, and these functions are linked to HSP activity.

It is already known that HS affects the immune response in pigs [50–52]. The genes found to overlap with the significant regions observed for T_ES_ include *BTBD17* (BTB domain containing 17), *PTPRE* (Protein Tyrosine Phosphatase Receptor Type E), and *ARNT2* (Aryl Hydrocarbon Receptor Nuclear Translocator 2). *BTBD17* has been reported to be involved in the negative regulation of viral genome replication and viral response [53, 54]. *PTPRE* has been shown to repress M2 macrophage activation and may have a critical role in regulating inflammatory responses and local homeostasis, particularly in the context of macrophage activation and function [55]. *ARNT2* regulates immune-related genes, phagocytic cell differentiation, and lymphocyte activity [56]. A polymorphism in *ARNT2* has been linked to reduced macrophage phagocytic activity, impacting immunity, platelet activation, and phagocytosis in pigs [56]. Additionally, we also found that the *NR1D1* (Nuclear Receptor Subfamily 1 Group D Member 1) gene harbors genomic markers associated with T_SS_ and T_RS_. *NR1D1* acts as a transcriptional repressor and is required for establishing and maintaining body temperature rhythm in a manner adaptable to environmental demands, allowing mammals to adapt to environmental temperature changes [57].

Previous studies have reported that some anatomical characteristics, such as the animal’s surface area, hair density, and body mass, can affect the ability of the animal to dissipate heat [23]. In this study we identified that regions associated with EA overlapping with the *HSP90AB1* (Heat Shock Protein 90 Alpha Family Class B Member 1) gene that encodes a member of the heat shock protein 90 family, as previously discussed. Additionally, we found that regions associated with HD overlap with the gene *DOCK10* (dedicator of cytokinesis 10). *DOCK10* is part of the dedicator of cytokinesis (DOCK) family proteins, which are expressed at varying levels in lymphocytes and have diverse effects on immune functions [58]. *DOCK10* influences the B cells development, which play a crucial role in the adaptive immune response by producing antibodies [58].

In the functional genomic analyses, the molecular functions of intracellular calcium activated chloride channel activity and chloride channel activity were found to be associated with T_ES_. The chloride anion plays a key role in regulating cellular homeostasis, affecting diverse cellular functions, including reactive oxygen species levels [59]. Oxidative stress stimulates calcium activated chloride channels [60–62], and promotes cellular and mitochondrial oxidative damage [63]. In addition, the molecular function of DNA replication origin binding was associate with T_SS_ and T_RS_ and the biological process of centrosome cycle was associated with T_TS_. These two GO terms are related to cell division and are affected by HS. HS can induce partial DNA re-replication and centrosome amplification in early S-phase cells [64]. Although HS causes centrosome degradation, HSP70 protects centrosome proteins from denaturation [65]. In the functional genomic analysis for RR, we identified two pathways: the pathway of *Staphylococcus aureus* infection and the pathway of complement and coagulation cascades. The latter is a proteolytic cascade in blood plasma and serves as a mediator of innate immunity, a nonspecific defense mechanism against pathogens [66–68].

### Candidate genes with pleiotropic effects for heat stress-related traits in lactating sows

Pleiotropy is a well-known effect that occurs when a locus influences more than one trait [16, 69]. In cases of pleiotropy, if a gene has effects on more than one trait, the genetic variation associated with one trait may be correlated with the genetic variation of another trait, resulting in a genetic correlation between the two traits. Freitas et al. [11] reported high genetic correlations among skin temperature traits (> 0.78) and among vaginal temperature traits (> 0.75). Similarly, high genetic correlations were observed for RR with PS (0.87), with BCS measures (0.92), and with ear length and ear area (0.95). Additionally, low to moderately positive genetic correlations were observed between skin temperatures and vaginal temperatures (ranging from 0.25 to 0.76). Low genetic correlations were estimated between vaginal temperatures and BCS (from − 0.01 to 0.06), while all other trait combinations were lowly genetically correlated [11].

Identifying candidate genes with pleiotropic effects is important to understand how a single gene can affect multiple traits and the common biological pathways involved in the expression of these traits. In GWAS studies, it is possible to identify the same regions or genes affecting different traits, which is termed cross-phenotype associations, however these associations might not be pleiotropy [16]. Solovieff et al. [16] defined three categories of pleiotropy: biological pleiotropy, mediated pleiotropy, and spurious pleiotropy. Biological pleiotropy refers to a genetic variant that directly influences more than one trait [16, 70]. Mediated pleiotropy is when a variant directly affects a first phenotype that affects the second phenotype [16, 70]. Spurious pleiotropy can occur due to some sources of bias, identifying genes that are not truly pleiotropic [16, 70].

In this study, to identify the pleiotropy among the traits, we grouped them as skin surface temperature traits (T_ES_, T_SS_, T_RS_, and T_TS_), vaginal temperatures (T_Vall_, T_V4days_, T_V8h_, T_V12h_, T_V16h_, and T_V20h_), animal body temperatures (i.e., all skin surface and vaginal temperature traits), respiration traits (RR and PS), thermoregulatory indicators (i.e., all skin surface temperatures, vaginal temperatures, and respiration traits), anatomical traits (BCS_cal_, BCS_vis_, HD, BS, EA, and EL), and a combination of all traits.

A common genomic region located on chromosome 7 was associated with vaginal temperature, animal body temperatures, and thermoregulatory traits groups. This region comprised the genes *RCAN2* (Regulator of Calcineurin 2), *ENPP5* (Ectonucleotide Pyrophosphatase/Phosphodiesterase Family Member 5), *ENPP4* (Ectonucleotide Pyrophosphatase/Phosphodiesterase 4), *CLIC5* (Chloride Intracellular Channel 5), and *U6* (U6 small nuclear RNA). *ENPP5* and *ENPP4* belong to the ectonucleotide pyrophosphatase/phosphodiesterase (ENPP) family of genes encoding a group of proteins that have enzymatic activity involved in the hydrolysis of nucleotides [71]. Based on their sequence homology, *ENPP4* and *ENPP5* have been described as putative ENPP ectoenzymes [72]. The ectoenzymes are found on leukocytes and endothelial cells and play a crucial role in regulating leukocyte trafficking [73]. *RCAN2* is a gene that encodes a protein involved in the regulation of the calcineurin signaling pathway. This protein is involved in many physiological processes by binding to the catalytic domain of calcineurin A and inhibiting calcineurin-mediated nuclear translocation of the transcription factor NFATC1 (nuclear factor of activated T cells 1) [74, 75]. Inactivation of the *RCAN2* gene in mice reduced age- and diet-induced obesity by causing a reduction in feed intake [76]. *CLIC5* encodes a member of the chloride intracellular channel (CLIC) family of chloride ion channels that regulates chloride transport and interacts with the cortical actin cytoskeleton in polarized epithelial cells [77]. *U6* is a small nuclear RNA (snRNA) involved in catalytic steps of pre-mRNA splicing [78]. This common genomic region identified for vaginal temperatures, animal body temperatures, and thermoregulatory traits groups might be relevant for thermoregulatory indicators since the genes annotated in this region are involved in immune response and in catalytic activities to reduce cell damage caused by oxidative stress.

The candidate genes *MCTP1* (Multiple C2 And Transmembrane Domain Containing 1), and *ELL2* (Elongation Factor for RNA Polymerase II 2), both located on chromosome 2, were candidate genes for animal temperature traits that also presented pleiotropic effects. *MCTP1* regulates endocytic recycling in central nervous system neurons and synapses, and possibly cellular vesicle retrieval and oxidative stress [79], which might influence HS response since it also causes cellular oxidative stress. *ELL2* has been reported to contribute to prostate homeostasis and potentially acts as a tumor suppressor in the development and progression of prostate cancer cells [80, 81]. Additionally, it regulates plasma cell gene expression, including immunoglobulin production in plasma cells [82]. Therefore, it is possible that the *ELL2* gene may influence immune response when the sows are under HS conditions.

We also annotated the protein-coding genes *NTN1* (Netrin-1) and *STX8* (Syntaxin 8) located on chromosome 12 for the thermoregulatory traits group. *NTN1* belongs to a family of laminin-related secreted proteins and contributes to the regulation of inflammation by influencing cell migration [83, 84]. *NTN1* is involved in immune response modulation, regulation of inflammation, and leukocyte infiltration [83]. *STX8* has been identified as a key player in the efficient sorting and trafficking of cytotoxic molecules to functional lytic granules in cytotoxic T lymphocytes in humans [85]. Both *NTN1* and *STX8* contribute significantly to the immune response, particularly in combating pathogens through the actions of macrophages and cytotoxic T cells.

For the RR group of traits, the gene *AKAP7* located on chromosome 1 was annotated. *AKAP7* is a gene encoding a protein kinase A-binding scaffolding molecule associated with a cellular regulatory pathway during viral infections [34, 35]. In addition, this gene promotes neuroprotection by enhancing mitochondrial networks and blocking apoptosis in glutamate-induced oxidative stress [36]. According to Gusho et al. [34], *AKAP7* may serve as a cytoprotective factor and potentially prevent tissue damage during viral infections. Several candidate genes with pleiotropic effects for physiological indicators of HS response in lactating sows were associated with immune response, supporting the already reported effects of HS on immune responses [50–52].

The gene *TMEM161B* (Transmembrane Protein 161B), located on chromosome 2, was found to be a candidate gene with pleiotropic effects for skin temperature traits. In zebrafish and mice, *TMEM161B* is linked with cardiac rhythm, preventing arrhythmic calcium oscillations, and ensuring proper blood flow for the developing embryo [86]. Moreover, studies indicate that increasing vasodilatation elevates skin blood flow, increasing skin temperature [87], which facilitates heat dissipation. In humans, *TMEM161B* has been reported to regulate cerebral cortical gyration, Sonic Hedgehog signaling, and ciliary structure in the developing central nervous system [88]. This gene might be important under HS conditions, where the sympathetic nervous system coordinates adjustments in cardiac rhythm and skin blood flow as response to thermal challenges [89, 90]. The rate of skin blood flow is primarily determined by body temperatures, and above an internal body temperature threshold for vasodilation, the increase in skin blood flow is proportional to the increase in internal temperature managing the impact of increased skin blood flow and heat transfer requirements on the circulatory system [90, 91].

For the anatomical traits group, the candidate genes with pleiotropic effects found included *ZMYND11* (Zinc finger MYND-type containing 11) and *DIP2C* (Disco Interacting Protein 2 homolog C) located on chromosome 10, and *GDNF* (Glial Cell Derived Neurotrophic Factor) and *CDH18* (Cadherin 18) on chromosome 16. *ZMYND11*, a multidomain protein, modulates RNA polymerase activity and regulates splicing. This gene was differentially regulated in response to thermal stress in sockeye and pink salmon [92] and in chicken pituitary under heat stress [93]. *ZMYND11* has been associated with neurodevelopmental disorders, global developmental delay, seizures, and hypotonia, and it is a potential causative gene in complex neurodevelopmental phenotypes [94–96]. While the biological functions of *DIP2C* are not entirely clear, it may regulate neuronal differentiation and early embryonic nervous development [97], as well as overall brain development and function [98]. *GDNF* is essential for maintaining neuronal morphological and neurochemical phenotypes, and protecting dopaminergic neurons from toxic damage [99]. It has several positive effects on the nervous system, including viability, proliferative activity, and migratory ability of cells [100]. *CDH18* has been associated with neuropsychiatric disorders in humans [101] and tumor suppressors [102, 103]. The candidate genes with pleiotropic effect for anatomical traits influence nervous system functions. The response to heat stress involves complex interactions between the brain and various neurotransmitter systems [104]. Furthermore, prolonged exposure to HS conditions can negatively affect central nervous system, leading to cognitive and neurological sequelae [105].

### Implications and limitations

Understanding the genetic background of physiological and anatomical traits related to HS response is valuable for breeding programs aimed to improve HS resilience in pigs. In this study we identified many genomic regions associated with indicators of HS response in lactating sows, but the QTL presented small effect sizes indicating that the traits are highly polygenic. The candidate genes for indicators of HS response evaluated are associated with catalytic activities to reduce cell damage from oxidative stress and cellular mechanisms related to immune response. Furthermore, gene ontology terms and pathways involved in heat shock protein activities, immune response, and oxidative cellular stress were found, which inform biological mechanisms related to heat tolerance and resilience in pigs. Additionally, in this study we identified variants with potential pleiotropic effects and influence in multiple indicators of HS response in lactating sows. This information allows breeders to design programs that could maximize genetic gain across multiple traits for more comprehensive improvements in animal populations. The utilization of imputed whole-genome sequencing (WGS) enabled the identification of significant variants that were absent on the medium-density SNP array. Additionally, pleiotropic variants can be filtered and included in the SNP array [106], which would benefit breeding programs focusing on selecting animals with greater climatic resilience. However, further investigations are required to assess pleiotropy between HS response indicators and traits related to production and reproduction. This evaluation is crucial to determine the importance of these variants for other relevant traits.

The use of crossbred animals represents a limitation of this study, as different genomic regions may be associated with the same indicators of HS response in purebred populations. This discrepancy arises because allelic frequencies vary across populations and breeds. Additionally, the dataset is not large, and the animals are from a single farm. Therefore, caution should be taken when extrapolating findings from crossbred animals to purebred populations, as the genetic background of the populations can significantly influence the observed associations between genomic regions and HS response indicators.

In future studies, the incorporation of economically important traits associated with reproductive and productive performance would allow the understanding of pleiotropy between these traits and those related to HS response. This expanded knowledge would provide valuable insights for creating selection indexes, enabling the identification of animals that are not only more resilient to HS but also capable of maintaining high productivity.

## Conclusions

In this study we identified numerous genomic regions significantly associated with indicators of HS response in lactating sows. The significant variants have small effects, indicating the highly polygenic nature of the traits evaluated. The candidate genes for physiological and anatomical indicators of HS response are associated with gene ontology terms and pathways involved in heat shock proteins activities, immune response, and oxidative cellular stress. The indicators of HS response exhibited pleiotropic effects, suggesting that multiple genomic regions influence various physiological and anatomical indicators of HS response. Many candidate genes with potential pleiotropic effects are associated with catalytic activities to reduce cell damage from oxidative stress and cellular mechanisms related to immune response were identified, providing valuable insights for breeding programs aimed at improving HS resilience in pigs. The findings have significant implications for advancing breeding programs aimed at improving heat tolerance in pigs.

## Materials and methods

### Animals and genotypes

The data used in this study was previously described by Johnson et al. [10] and Freitas et al. [11]. In summary, the phenotypes were collected from 1,645 multiparous lactating sows (Large White x Landrace cross) at a commercial sow farm located in Maple Hill, North Carolina, USA. A total of 1,639 sows were genotyped using the PorcineSNP50K (50,703 SNPs) Bead Chip (Illumina, San Diego, CA, USA). The quality control applied to the genotypes consisted of removing animals with a call rate lower than 0.80 and SNPs with a call rate lower than 0.90, and SNPs located on non-autosomal chromosomes. After quality control, 36,258 SNPs for 1,622 animals remained for further analyses. The genotypes were imputed to WGS containing 34,615,361 SNPs using the Swine Imputation (SWIM) Server [17]. The reference population consisted of 2,259 animals from various breeds, including 615 Landrace and 543 Large White, and 25 Landrace x Large White crossbred animals [17].

After genotype imputation, additional quality control was performed, removing variants with impute scores lower than 0.85, animals and SNPs with missing rates greater than 0.10, minor allele frequency (MAF) lower than 0.05, and extreme *p*-values for Hardy-Weinberg equilibrium tests lower than 1 × 10^− 15^. Data from 7,065,922 SNPs for 1,622 animals remained for subsequent analyses. LD-based pruning was performed considering a pairwise correlation coefficient threshold of 0.90 between markers, and a window size of 10 markers, with a step size of five markers, retaining 1,710,312 SNPs. Some advantages of performing LD pruning are to reduce redundancy and confounding factors that may interfere with statistical analyses and reduce the number of tests performed improving the power of the analyses [107]. Genotyping data was pre-processed using the Plink software [108].

### Phenotypic records

The description of the phenotypes evaluated in this study are presented in Table 1. In summary, the thermoregulatory indicators of HS response phenotypes collected were respiration rate (RR), ear skin temperature (T_ES_), shoulder skin temperature (T_SS_), rump skin temperature (T_RS_) tail skin temperature (T_TS_), vaginal temperatures, and panting score (PS). RR and skin surface temperatures were collected at 0800, 1200, 1600, and 2000 h daily during a period of four consecutive days. Vaginal temperature was automatically recorded in 10 min intervals over four days using calibrated thermochron temperature recorders. This data was used to compute six traits in this study: whole data measured at every 10 min (T_Vall_), the average of the six records per hour corresponding to 0800, 1200, 1600, and 2000 h during four days (T_V4days_), and single record corresponding to 0800, 1200, 1600, and 2000 h measured at the first day of collection (T_V8h_, T_V12h_, T_V16h_, and T_V20h_, respectively). Panting score was collected during four consecutive days at 15:30 h and scored from 0 to 3 (0: if it was with closed mouth and normal breathing; 1: closed mouth and rapid breathing; 2: open mouth and rapid breathing; 3: open mouth and rapid breathing with obvious salivation).

We also collected phenotypes for sow anatomical traits that might influence on the ability of the animal to dissipate metabolic heat. The traits measured include hair density (HD), body condition score (BCS), body size (BS), ear length (EL), and ear area (EA). HD was measured as a subjective visual score ranging from 0 to 2, being the score 0 = hairless or limited hair cover, 1 = normal or moderate hair cover, and 2 = sow with greater than normal hair cover. BCS was evaluated using a sow caliper (BCS_cal_) [109] and it was also evaluated as a visual BCS (BCS_vis_) considering five categories: 1 = emaciated, 2 = thin, and 3 = ideal, 4 = fat, and 5 = overly fat. The animals were also score into three categories according to their BS as small, medium, or large. The descriptive statistics for the traits used in this study are presented in Table 4. More details about the data collection procedures are presented in Johnson et al. [10].

**Table 4:** Descriptive statistics for continuous heat stress indicators in Landrace x Large White crossbred pig population

Trait^1^	Number of animals	Number of observations	Mean	Minimum	Maximum	Coefficient of variation (%)
T_ES_	1,381	21,411	36.70	32.50	40.70	2.92
T_SS_	1,381	21,414	36.50	32.30	39.80	2.96
T_RS_	1,381	21,414	37.20	33.60	39.90	2.47
T_TS_	1,381	21,413	36.90	33.20	40.00	2.57
RR	1,381	21,360	73.00	12.00	172.00	38.74
PS	1,380	5,450	1.02	0	3	58.82
T_Vall_	1,381	932,708	39.74	37.08	42.35	1.91
T_V4days_	1,381	21,415	39.70	37.10	42.70	1.94
T_V8h_	1,357	1,357	39.25	37.43	41.43	1.53
T_V12h_	1,370	1,370	39.69	37.08	41.61	1.61
T_V16h_	1,354	1,354	40.15	37.35	42.72	1.72
T_V20h_	1,362	1,362	40.25	37.85	41.84	1.71
HD	1,151	1,151	2.04	1	3	31.37
BS	1,379	1,379	2.26	1	3	27,43
BCS_cal_	1,361	1,361	6.93	1	10	29.15
BCS_vis_	1,348	1,348	2.10	1	3	30.48
EA	705	705	309.01	183.23	487.86	17.37
EL	713	713	24.98	14.83	34.34	11,25

^1^T_ES_: ear skin temperature (°C); T_SS_: shoulder skin temperature (°C); T_RS_: rump skin temperature (°C); T_TS_: tail skin temperature (°C); RR: respiration rate (breaths per minute); PS: panting score; T_Vall_: all measures (every 10 min) of vaginal temperatures (°C); T_V4days_: average of the six records per hour corresponding to 08:00, 12:00, 16:00, and 20:00 h during four days (°C); T_V8h_: vaginal temperature measured on the first day at 8:00 (°C); T_V12h_: vaginal temperature measured on the first day at 12:00 (°C); T_V16h_: vaginal temperature measured on the first day at 16:00 (°C); T_V16h_: vaginal temperature measured on the first day at 20:00 (°C); HD: hair density score; BS: body size; BCS_cal_: body condition score using a sow caliper; BCS_vis_: visual body condition score; EA: ear area (cm^2^); EL: and ear length (cm). Data collection protocols have been described by Johnson et al. [10] and Freitas et al. [11]

The phenotypes were pre-corrected for fixed effects to be used as input for the GWAS analyses. The fixed effects for each trait were described by Freitas et al. [11]. In summary, the fixed effects fitted for skin surface temperature (T_ES_, T_SS_, T_RS_, and T_TS_) and RR were trait recorder; concatenation of week, day, and time of measurement; parity; days in lactation; concatenation of barn type and room; and in-barn environmental temperature as a linear covariate. For PS, the fixed effects were trait recorder; concatenation of week and day of measurement; parity; days in lactation; concatenation of barn type and room; and in-barn environmental temperature as a linear covariate. For T_Vall_, the fixed effects were the concatenation of week and day of measurement; parity; concatenation of barn type and room; and in-barn environmental temperature as a linear covariate. For T_V4days_, the fixed effects were concatenation of week, day, and time of measurement; parity; days in lactation; concatenation of barn type and room; and in-barn environmental temperature as a linear covariate. For T_V8h_, T_V12h_, T_V16h_, and T_V20h_, the fixed effects were the concatenation of week and day of measurement; parity; days in lactation; concatenation of barn type and room; and in-barn environmental temperature as a linear covariate. For HD, the fixed effects fitted were trait recorder and parity. For BS, the fixed effects fitted were trait recorder, week of measurement, and parity. For BCS_cal_ and BCS_vis_, the fixed effect fitted were trait recorder, week of measurement, parity, days in lactation, and concatenation of barn type and room. For EA and EL, the fixed effects fitted were trait recorder and picture quality. For traits that presented repeated measurements (i.e., the thermoregulatory traits: skin surface temperatures, vaginal temperatures, RR, and PS), we computed the average adjusted phenotype to be used as response variables for GWAS [110].

### Genome-wide association studies

The GWAS was performed considering the WGS dataset using the leave-one-chromosome-out approach on mixed linear model association analysis (MLMA-LOCO) implemented in the GCTA software [111]. The model can be described as:

$${\bf{y}} = {\bf{1}}\mu + {\bf{X}}{\rm{b}} + {\bf{Zu}} + \varepsilon ,$$,

Where $\mathbf{y}$ is the vector of pre-corrected phenotypes for each analyzed trait; ${\bf{1}}$ is a vector of ones; ${\mu }$ is the overall mean; $\text{b}$ is the fixed effect of each tested SNP; $\mathbf{X}$ is a vector containing the genotype for SNP; $\mathbf{u}$ is the random vector of polygenic effect with $\mathbf{u} \sim \text{N}(0,\mathbf{G}{{\sigma }}_{u}^{2})$, where $\mathbf{G}$ is the genomic-based relationship matrix (GRM) as previously described [111], ${{\sigma }}_{u}^{2}$ is the additive genetic variance; $\mathbf{Z}$ is the incidence matrix for $\mathbf{u}$; and $\varvec{\epsilon }$ is a random vector of residual effects with $\varvec{\epsilon } \sim \text{N}(0,\mathbf{I}{{\sigma }}_{\epsilon }^{2})$, where $\mathbf{I}$ is an identity matrix and ${{\sigma }}_{\epsilon }^{2}$ is the residual variance.

The MLMA-LOCO approach is based on fitting a different genomic relationship matrix (GRM) to account for population stratification for the association tests of each chromosome by excluding the markers located on the chromosome where the tested SNP are located when creating the GRM for that chromosome [112]. The inclusion of the marker being tested in the GRM can reduce the statistical test power due to the double fitting of the marker in the model: both as a fixed effect for association and as a random effect as part of the GRM [112–114]. This phenomenon was termed “proximal contamination” by Listgarten et al. [113], who demonstrated that a mixed linear model excluding the marker from GRM is the mathematically correct approach and provided an efficient algorithm for mixed linear model analysis.

### Adjustment for multiple testing

To check if there was potential population stratification due to the population structure, the genomic inflation factor (λ) [115] was evaluated, along with its 95% confidence interval. Due to the multiple testing nature of the GWAS method employed in this study, there is a cumulative probability of false-positive associated SNPs [116, 117]. In this sense, a Bonferroni correction at α = 0.05 for the genome-wise significance level [118] was applied, by dividing α by the number of independent chromosome segments ($Me$) to account for the dependence among tests due to LD. The $Me$ was calculated as proposed by Goddard et al. [119]:

$$Me= \frac{2NeLk}{\text{l}\text{o}\text{g}\left(NeL\right)},$$

where $Ne$ is the effective population size, which was computed as 85 according to populational LD [120]; $L$ is the average length of chromosome expressed in Morgan; and $k$ is the number of chromosomes. A SNP was considered as statistically significant if its *p*-value was smaller than 0.05/$Me$, which was equal to 1.21 × 10^− 6^.

### Pleiotropic effects

The multiple-trait analysis proposed by Bolormaa et al. [18] allows to evaluate the pleiotropy in groups of more than two traits. This approach is based on combining the results from the single-trait association analyses to calculate a multi-trait statistic and it has been demonstrated that the multi-trait approach proposed by them has a lower FDR than a single trait analysis (at the same significance test *p*-value) [18, 121]. First, the phenotypes were Cholesky-decorrelated because the Bolormaa et al. [18] approach was originally proposed to perform meta-analysis, i.e., independent studies [19, 20]. Then, the traits were grouped in seven different categories: skin surface temperature traits (i.e., T_ES_, T_SS_, T_RS_, and T_TS_), vaginal temperatures (i.e., T_Vall_, T_V4days_, T_V8h_, T_V12h_, T_V16h_, and T_V20h_), animal temperatures (all skin surface and vaginal temperature traits), respiration traits (i.e., RR and PS), thermoregulatory indicators (i.e., all skin surface and vaginal temperature traits, RR and PS), anatomical traits (i.e., BCS_cal_, BCS_vis_, HD, BS, EA, and EL), and all traits to evaluate the presence of pleiotropic effects in each trait group. The null hypothesis tested states that the SNP does not affect any of the $n$ traits in the group based on the ${\chi }^{2}$ with $n$ degrees of freedom. The multi-trait statistics for each SNP was calculated as presented by [18]:

$${\rm{Multi - trait}}\,{\chi ^2} = t_i^\prime {V^{ - 1}}{t_i},$$

where ${\varvec{t}}_{i}$ is a $n$ x 1 vector of signed t-values for the ${i}^{th}$ SNP across the $n$ trait, ${\varvec{t}}_{i}^{{\prime }}$ is the transpose of ${\varvec{t}}_{i}$, and ${\varvec{V}}^{-1}$ is the inverse of the $n$ x $n$ correlation matrix over all estimated SNP effects. As this method presents a distribution associated to the ${\chi }^{2}$ statistics, each marker presents a statistic and a *p*-value to infer if the marker is significant or not for pleiotropic effects for the group of traits evaluated. A Bonferroni correction was also applied in the same way described for the GWAS, then a variant was considered with significant pleiotropic effect if its *p*-value was smaller than 1.21 × 10^− 6^.

### Gene annotation and functional analyses

Gene annotation was performed within a region of ± 100 kb around the significant SNPs using the GALLO package [122]. The annotated data for *Sus scrofa* from the Ensembl database (www.ensembl.org/Sus_scrofa/Info/Index) were used in the analyses. The web-based tool Database for Annotation, Visualization, and Integrated Discovery (DAVID) [123, 124] was used for conducting Gene Ontology (GO) and KEGG pathway enrichment (*P* < 0.05) analyses to identify biological processes, molecular functions, cellular components, and biological pathways associated with the positional candidate genes identified. DAVID is a web-based tool for gene-enrichment analysis that utilizes a knowledge base aggregating information from various heterogeneous and widely distributed public databases [124].

### Electronic supplementary material

Below is the link to the electronic supplementary material.

Supplementary Material 1

Supplementary Material 2

Supplementary Material 3

Supplementary Material 4

## Data Availability

The dataset can be made available for research purposes by contacting Dr. Luiz Brito (britol@purdue.edu).

## References

[1] NOAA National Centers for Environmental Information. Monthly global climate report for annual 2022. 2023.

[2] Johnson JS (2018). Heat stress: impact on livestock well-being and productivity and mitigation strategies to alleviate the negative effects. Anim Prod Sci.

[3] Ingram DL, Monteith JL, Mount LE. Heat loss and its control in pigs. Heat loss Anim man Assess Control. 1973:235–54.

[4] Renaudeau D, Collin A, Yahav S, De Basilio V, Gourdine JL, Collier RJ. Adaptation to hot climate and strategies to alleviate heat stress in livestock production. In: Animal. 2012. p. 707–28. 10.1017/S1751731111002448 22558920

[5] Misztal I. Breeding and genetics symposium: resilience and lessons from studies in genetics of heat stress. J Anim Sci. 2017;95:1780–7. 10.2527/jas.2016.0953 28464095

[6] Tiezzi F, Brito LF, Howard J, Huang YJ, Gray K, Schwab C et al. Genomics of heat tolerance in reproductive performance investigated in four independent maternal lines of pigs. Front Genet. 2020;11. 10.3389/fgene.2020.00629 PMC7338773 32695139

[7] Freitas PHF, Johnson JS, Chen S, Oliveira HR, Tiezzi F, Lázaro SF et al. Definition of environmental variables and critical periods to evaluate heat tolerance in large white pigs based on single-step genomic reaction norms. Front Genet. 2021;12. 10.3389/fgene.2021.717409 PMC8650309 34887897

[8] Dodd GR, Gray K, Huang Y, Fragomeni B. Single-step GBLUP and GWAS analyses suggests implementation of unweighted two trait approach for heat stress in swine. Animals. 2022;12. 10.3390/ani12030388 PMC8833662 35158711

[9] Ravagnolo O, Misztal I, Hoogenboom G (2000). Genetic component of heat stress in dairy cattle, development of heat index function. J Dairy Sci.

[10] Johnson JS, Wen H, Freitas PHF, Maskal JM, Hartman SO, Byrd MK (2023). Evaluating phenotypes associated with heat tolerance and identifying moderate and severe heat stress thresholds in lactating sows housed in mechanically or naturally ventilated barns during the summer under commercial conditions. J Anim Sci.

[11] Freitas PHF, Johnson JS, Wen H, Maskal JM, Tiezzi F, Maltecca C (2023). Genetic parameters for automatically-measured vaginal temperature, respiration efficiency, and other thermotolerance indicators measured on lactating sows under heat stress conditions. Genet Selection Evol.

[12] Visscher PM, Brown MA, McCarthy MI, Yang J (2012). Five years of GWAS discovery. Am J Hum Genet.

[13] Freitas PHF, Johnson JS, Tiezzi F, Huang Y, Schinckel AP, Brito LF (2023). Genomic predictions and GWAS for heat tolerance in pigs based on reaction norm models with performance records and data from public weather stations considering alternative temperature thresholds. J Anim Breed Genet.

[14] Kim K-S, Seibert JT, Edea Z, Graves KL, Kim E-S, Keating AF et al. Characterization of the acute heat stress response in gilts: III. Genome-wide association studies of thermotolerance traits in pigs RUNNING HEAD: genetic control of heat stress response in gilts. 2018. 10.1093/jas/sky131/4970896 10.1093/jas/sky131 PMC6095244 29669012

[15] Stearns FW (2010). One hundred years of Pleiotropy: a retrospective. Genetics.

[16] Solovieff N, Cotsapas C, Lee PH, Purcell SM, Smoller JW (2013). Pleiotropy in complex traits: challenges and strategies. Nat Rev Genet.

[17] Ding R, Savegnago R, Liu J, Long N, Tan C, Cai G et al. The SWine IMputation (SWIM) haplotype reference panel enables nucleotide resolution genetic mapping in pigs. Communications Biology 2023 6:1. 2023;6:1–10. 10.1038/s42003-023-04933-9 PMC10229620 37253973

[18] Bolormaa S, Pryce JE, Reverter A, Zhang Y, Barendse W, Kemper K et al. A multi-trait, meta-analysis for detecting pleiotropic polymorphisms for stature, fatness and reproduction in beef cattle. PLoS Genet. 2014;10. 10.1371/journal.pgen.1004198 PMC3967938 24675618

[19] Xiang R, van den Berg I, MacLeod IM, Daetwyler HD, Goddard ME. Effect direction meta-analysis of GWAS identifies extreme, prevalent and shared pleiotropy in a large mammal. Communications Biology 2020 3:1. 2020;3:1–14. 10.1038/s42003-020-0823-6 PMC7048789 32111961

[20] Xiang R, MacLeod IM, Bolormaa S, Goddard ME (2017). Genome-wide comparative analyses of correlated and uncorrelated phenotypes identify major pleiotropic variants in dairy cattle. Sci Rep 2017.

[21] Collier RJ, Baumgard LH, Zimbelman RB, Xiao Y (2019). Heat stress: physiology of acclimation and adaptation. Anim Front.

[22] Ross JW, Hale BJ, Gabler NK, Rhoads RP, Keating AF, Baumgard LH (2015). Physiological consequences of heat stress in pigs. Anim Prod Sci.

[23] Sejian V, Bhatta R, Gaughan JB, Dunshea FR, Lacetera N (2018). Review: adaptation of animals to heat stress. Animal.

[24] Shin J, Lee C (2015). Statistical power for identifying nucleotide markers associated with quantitative traits in genome-wide association analysis using a mixed model. Genomics.

[25] Khanzadeh H, Ghavi Hossein-Zadeh N, Ghovvati S (2022). The statistical power of genome-wide association studies for threshold traits with different frequencies of causal variants. Genetica.

[26] Sanchez MP, Govignon-Gion A, Croiseau P, Fritz S, Hozé C, Miranda G (2017). Within-breed and multi-breed GWAS on imputed whole-genome sequence variants reveal candidate mutations affecting milk protein composition in dairy cattle. Genet Selection Evol.

[27] Daetwyler HD, Capitan A, Pausch H, Stothard P, Van Binsbergen R, Brøndum RF (2014). Whole-genome sequencing of 234 bulls facilitates mapping of monogenic and complex traits in cattle. Nat Genet.

[28] Van Den Berg S, Vandenplas J, Van Eeuwijk FA, Bouwman AC, Lopes MS, Veerkamp RF (2019). Imputation to whole-genome sequence using multiple pig populations and its use in genome-wide association studies. Genet Selection Evol.

[29] Wu Y, Zheng Z, Visscher PM, Yang J (2017). Quantifying the mapping precision of genome-wide association studies using whole-genome sequencing data. Genome Biol.

[30] Yang J, Weedon MN, Purcell S, Lettre G, Estrada K, Willer CJ (2011). Genomic inflation factors under polygenic inheritance. Eur J Hum Genet 2011.

[31] Hayes B, Gondro C, van der Werf J, Hayes B (2013). Overview of statistical methods for Genome-Wide Association Studies (GWAS). Genome-Wide Association Studies and genomic prediction.

[32] Lapinski PE, Kwon S, Lubeck BA, Wilkinson JE, Srinivasan RS, Sevick-Muraca E (2012). RASA1 maintains the lymphatic vasculature in a quiescent functional state in mice. J Clin Invest.

[33] He X, Howard BA, Liu Y, Squillace DL, Klein BS, Lawrence CB (2021). LYSMD3: a mammalian pattern recognition receptor for chitin ll LYSMD3: a mammalian pattern recognition receptor for chitin. CellReports.

[34] Gusho E, Jha BK, Zhang R, Weiss SR, Silverman H (2013). Preventing activation of the IFN inducible OAS-RNase L pathway by A-kinase anchoring protein 7 (AKAP7). Cytokine.

[35] King CR, Cohen MJ, Fonseca GJ, Dirk BS, Dikeakos JD, Mymryk JS (2016). Functional and Structural Mimicry of Cellular protein kinase A anchoring proteins by a viral oncoprotein. PLoS Pathog.

[36] Zhang J, Feng J, Ma D, Wang F, Wang Y, Li C (2019). Neuroprotective mitochondrial remodeling by AKAP121/PKA protects HT22 cell from Glutamate-Induced oxidative stress. Mol Neurobiol.

[37] Wen H, Johnson JS, Freitas PHF, Maskal JM, Gloria LS, Araujo AC (2023). Longitudinal genomic analyses of automatically-recorded vaginal temperature in lactating sows under heat stress conditions based on random regression models. Genet Selection Evol.

[38] Richter K, Haslbeck M, Buchner J (2010). The heat shock response: life on the verge of death. Mol Cell.

[39] Anckar J, Sistonen L. Regulation of HSF1 function in the heat stress response: implications in aging and disease. 2011;80:1089–115.https://doi.org/101146/annurev-biochem-060809-095203 10.1146/annurev-biochem-060809-095203 21417720

[40] Li GC, Li L, Liu RY, Rehman M, Lee WMF. Heat shock protein hsp70 protects cells from thermal stress even after deletion of its ATP-binding domain. Proceedings of the National Academy of Sciences. 1992;89:2036–40. 10.1073/pnas.89.6.2036 PMC48591 1549562

[41] Beere HM (2001). Stressed to death: regulation of apoptotic signaling pathways by the heat shock proteins. Science’s STKE.

[42] Chatterjee BK, Puri S, Sharma A, Pastor A, Chaudhuri TK, Chatterjee BK et al. Molecular chaperones: structure-function relationship and their role in protein folding. 2018:181–218.

[43] Dangi SS, Bharati J, Samad HA, Kumar Bhure S, Singh G, Prakash Maurya V (2017). Expression Dynamics of Heat Shock proteins (HSP) in livestock under thermal stress. Heat Shock Proteins.

[44] Calderwood SK, Gong J, Murshid A. Extracellular HSPs: the complicated roles of extracellular HSPs in immunity. Front Immunol. 2016;7 APR:187592. 10.3389/fimmu.2016.00159 PMC4842758 27199984

[45] Chen K, He Y, Liu Y, Yang X (2019). Gene signature associated with neuro-endocrine activity predicting prognosis of pancreatic carcinoma. Mol Genet Genomic Med.

[46] Haase M, Fitze G (2016). HSP90AB1: helping the good and the bad. Gene.

[47] Brown-Brandl TM, Eigenberg RA, Nienaber JA, Kachman SD (2001). Thermoregulatory profile of a newer genetic line of pigs. Livest Prod Sci.

[48] Gourdine JL, Rauw WM, Gilbert H, Poullet N. The genetics of thermoregulation in pigs: a review. Front Veterinary Sci. 2021;8. 10.3389/fvets.2021.770480 PMC8711629 34966808

[49] Archana P, Aleena J, Pragna P, Vidya M, Abdul Niyas P, Bagath M (2017). Role of heat shock proteins in livestock adaptation to heat stress. J Dairy Veterinary Anim Res.

[50] Cui Y, Wang C, Hao Y, Gu X, Wang H. Chronic heat stress induces acute phase responses and serum metabolome changes in finishing pigs. Animals. 2019;9. 10.3390/ani9070395 PMC6680871 31261803

[51] Johnson JS, Maskal JM, Duttlinger AW, Kpodo KR, McConn BR, Byrd CJ (2020). In utero heat stress alters the postnatal innate immune response of pigs. J Anim Sci.

[52] Yu TY, Yong YH, Li JY, Fang B, Hu CY, Wu LY et al. Proteomic study of hypothalamus in pigs exposed to heat stress. BMC Vet Res. 2020;16. 10.1186/s12917-020-02505-1 PMC7424663 32787853

[53] Logsdon BA, Perumal TM, Daily K, Sieberts SK, Omberg L, Mangravite LM, STUDIES IMPLICATE GENES INVOLVED IN GLIAL CELL FUNCTION AND VIRAL RESPONSE IN CEREBRAL WHITE MATTER HYPERINTENSITIES (2018). O3-03-03: EPIGENOME-WIDE ASSOCIATION. Alzheimer’s Dement.

[54] Chung O, Jung YE, Lee KW, An YJ, Kim J, Roh YR (2022). The analyses of Cetacean Virus-responsive genes reveal Evolutionary Marks in Mucosal Immunity-Associated genes. Biochem Genet.

[55] Gerhart-Hines Z, Feng D, Emmett MJ, Everett LJ, Loro E, Briggs ER (2013). The nuclear receptor Rev-erbα controls circadian thermogenic plasticity. Nature.

[56] Crespo-Piazuelo D, Ramayo-Caldas Y, González-Rodríguez O, Pascual M, Quintanilla R, Ballester M (2021). A Co-association Network Analysis reveals putative regulators for Health-related traits in pigs. Front Immunol.

[57] Gerhart-Hines Z, Feng D, Emmett MJ, Everett LJ, Loro E, Briggs ER (2013). The nuclear receptor Rev-erbα controls circadian thermogenic plasticity. Nat 2013.

[58] Chen Y, Chen Y, Yin W, Han H, Miller H, Li J (2021). The regulation of DOCK family proteins on T and B cells. J Leukoc Biol.

[59] Valdivieso ÁG, Santa-Coloma TA (2019). The chloride anion as a signalling effector. Biol Rev.

[60] Averaimo S, Milton RH, Duchen MR, Mazzanti M (2010). Chloride intracellular channel 1 (CLIC1): Sensor and effector during oxidative stress. FEBS Lett.

[61] Wills NK, Weng T, Mo L, Hellmich H, Yu A, Wang T et al. Chloride channel expression in cultured human fetal RPE cells: response to oxidative stress. Invest Ophthalmol Vis Sci. 41 13:4247–55. 11095622

[62] Jeulin C, Guadagnini R, Marano F (2005). Oxidant stress stimulates Ca2+-activated chloride channels in the apical activated membrane of cultured nonciliated human nasal epithelial cells. Am J Physiol Lung Cell Mol Physiol.

[63] Belhadj Slimen I, Najar T, Ghram A, Abdrrabba M (2016). Heat stress effects on livestock: molecular, cellular and metabolic aspects, a review. J Anim Physiol Anim Nutr (Berl).

[64] Petrova NV, Velichko AK, Razin SV, Kantidze OL (2016). Early S-phase cell hypersensitivity to heat stress. Cell Cycle.

[65] Vertii A, Zimmerman W, Ivshina M, Doxsey S (2015). Centrosome-intrinsic mechanisms modulate centrosome integrity during fever. Mol Biol Cell.

[66] Oikonomopoulou K, Ricklin D, Ward PA, Lambris JD (2012). Interactions between coagulation and complement - their role in inflammation. Semin Immunopathol.

[67] Mathern DR, Heeger PS (2015). Molecules great and small: the complement system. Clin J Am Soc Nephrol.

[68] Bajic G, Degn SE, Thiel S, Andersen GR (2015). Complement activation, regulation, and molecular basis for complement-related diseases. EMBO J.

[69] Paaby AB, Rockman MV (2013). The many faces of pleiotropy. Trends Genet.

[70] Hackinger S, Zeggini E. Statistical methods to detect pleiotropy in human complex traits. Open Biol. 2017;7. 10.1098/rsob.170125 PMC5717338 29093210

[71] Borza R, Salgado-Polo F, Moolenaar WH, Perrakis A (2022). Structure and function of the ecto-nucleotide pyrophosphatase/ phosphodiesterase (ENPP) family: tidying up diversity. J Biol Chem.

[72] Gijsbers R, Ceulemans H, Stalmans W, Bollen M. Structural and catalytic similarities between nucleotide pyrophosphatases/phosphodiesterases and alkaline phosphatases. 2000. 10.1074/jbc.M007552200 10.1074/jbc.M007552200 11027689

[73] Salmi M, Jalkanen S (2012). Ectoenzymes controlling leukocyte traffic. Eur J Immunol.

[74] Serrano-Candelas E, Farré D, Aranguren-Ibáñez Á, Martínez-Høyer S, Pérez-Riba M (2014). The Vertebrate RCAN Gene Family: Novel insights into Evolution, structure and regulation. PLoS ONE.

[75] Davies KJA, Ermak G, Rothermel BA, Pritchard M, Heitman J, Ahnn J (2007). Renaming the DSCR1 / Adapt78 gene family as RCAN: regulators of calcineurin. FASEB J.

[76] Sun XY, Hayashi Y, Xu S, Kanou Y, Takagishi Y, Tang YP (2011). Inactivation of the Rcan2 gene in mice ameliorates the age- and Diet-Induced obesity by causing a reduction in Food Intake. PLoS ONE.

[77] Berryman M, Bretscher A (2000). Identification of a novel member of the chloride intracellular channel gene family (CLIC5) that associates with the actin cytoskeleton of placental microvilli. Mol Biol Cell.

[78] Kandels-Lewis S, Séraphin B. Role of U6 snRNA in 5’ splice site selection. Science (1979). 1993;262:2035–9. 10.1126/science.8266100 8266100

[79] Qiu L, Yu H, Liang F (2015). Multiple C2 domains transmembrane protein 1 is expressed in CNS neurons and possibly regulates cellular vesicle retrieval and oxidative stress. J Neurochem.

[80] Pascal LE, Masoodi KZ, Liu J, Qiu X, Song Q, Wang Y (2017). Conditional deletion of ELL2 induces murine prostate intraepithelial neoplasia. J Endocrinol.

[81] Wang Z, Pascal LE, Chandran UR, Chaparala S, Lv S, Ding H (2020). ELL2 is required for the growth and survival of AR-Negative prostate Cancer cells. Cancer Manag Res.

[82] Ghobrial A, Flick N, Daly R, Hoffman M, Milcarek C. ELL2 influences transcription elongation, splicing, Ig secretion and growth. J Mucosal Immunol Res. 2019;3. PMC6953911 31930204

[83] Xia X, Hu Z, Wang S, Yin K (2022). Netrin-1: an emerging player in inflammatory diseases. Cytokine Growth Factor Rev.

[84] Ziegon L, Schlegel M (2021). Netrin-1: a modulator of Macrophage Driven Acute and chronic inflammation. Int J Mol Sci 2022.

[85] Bhat SS, Friedmann KS, Knörck A, Hoxha C, Leidinger P, Backes C (2016). Syntaxin 8 is required for efficient lytic granule trafficking in cytotoxic T lymphocytes. Biochimica et Biophysica Acta (BBA). Mol Cell Res.

[86] Koopman CD, de Angelis J, Iyer SP, Verkerk AO, Silva J, Da, Berecki G et al. The zebrafish grime mutant uncovers an evolutionarily conserved role for Tmem161b in the control of cardiac rhythm. Proceedings of the National Academy of Sciences. 2021;118. 10.1073/pnas.2018220118 PMC7936323 33597309

[87] Chou T-H, Coyle EF. Cardiovascular responses to hot skin at rest and during exercise. 2022. 10.1080/23328940.2022.2109931 10.1080/23328940.2022.2109931 PMC10405766 37554384

[88] Akula SK, Marciano JH, Lim Y, Exposito-Alonso D, Hylton NK, Hwang GH et al. TMEM161B regulates cerebral cortical gyration, sonic hedgehog signaling, and ciliary structure in the developing central nervous system. Proc Natl Acad Sci U S A. 2023;120. 10.1073/pnas.2209964120 PMC9942790 36669111

[89] Charkoudian N. Skin blood flow in adult human thermoregulation: how it works, when it does not, and why. Mayo Clin Proc. 2003;78:603–12. 10.4065/78.5.603 12744548

[90] Ootsuka Y, Tanaka M (2015). Control of cutaneous blood flow by central nervous system. Temperature.

[91] Nadel ER, Cafarelli E, Roberts MF, Wenger CB (1979). Circulatory regulation during exercise in different ambient temperatures. J Appl Physiol Respir Environ Exerc Physiol.

[92] Akbarzadeh A, Günther OP, Houde AL, Li S, Ming TJ, Jeffries KM (2018). Developing specific molecular biomarkers for thermal stress in salmonids. BMC Genomics.

[93] Pritchett EM, Van Goor A, Schneider BK, Young M, Lamont SJ, Schmidt CJ (2023). Chicken pituitary transcriptomic responses to acute heat stress. Mol Biol Rep.

[94] Tumiene B, Čiuladaitė, Preikšaitienė E, Mameniškienė R, Utkus A, Kučinskas V (2017). Phenotype comparison confirms ZMYND11 as a critical gene for 10p15.3 microdeletion syndrome. J Appl Genet.

[95] Moskowitz AM, Belnap N, Siniard AL, Szelinger S, Claasen AM, Richholt RF (2016). A de novo missense mutation in ZMYND11 is associated with global developmental delay, seizures, and hypotonia. Cold Spring Harb Mol Case Stud.

[96] Yates TM, Drucker M, Barnicoat A, Low K, Gerkes EH, Fry AE (2020). ZMYND11-related syndromic intellectual disability: 16 patients delineating and expanding the phenotypic spectrum. Hum Mutat.

[97] Yao M, Su P, Li Z, Cui X, Yang Q, Xing X (2021). Knockout of Dip2c in murine ES cell line IBMSe001-B-1 by CRISPR/Cas9 genome editing technology. Stem Cell Res.

[98] Oo ZM, Adlat S, Sah RK, Myint MZZ, Hayel F, Chen Y (2020). Brain transcriptome study through CRISPR/Cas9 mediated mouse Dip2c gene knock-out. Gene.

[99] De Tassigny XD, anglemont, Pascual A, Lopez-Barneo J (2015). GDNF-based therapies, GDNF-producing interneurons, and trophic support of the dopaminergic nigrostriatal pathway. Implications for parkinson’s disease. Front Neuroanat.

[100] Revishchin AV, Parshina VV, Pavlova GV (2022). [The role of glial cell line-derived neurotrophic factor isoforms in human glial tumors]. Zh Vopr Neirokhir Im N N Burdenko.

[101] Redies C, Hertel N, Hübner CA (2012). Cadherins and neuropsychiatric disorders. Brain Res.

[102] Bai YH, Zhan YB, Yu B, Wang WW, Wang L, Zhou JQ (2018). A novel Tumor-Suppressor, CDH18, inhibits Glioma Cell Invasiveness Via UQCRC2 and correlates with the prognosis of Glioma patients. Cell Physiol Biochem.

[103] Zhao B, Wu J, Cha X, Mao G, Shi H, Fei S et al. Effect of COP1 in promoting the tumorigenesis of gastric cancer by down-regulation of CDH18 via PI3K/AKT signal pathway. Anal Cell Pathol (Amst). 2023;2023. 10.1155/2023/5617875 PMC10072965 37025097

[104] Nybo L (2007). Exercise and heat stress: cerebral challenges and consequences. Prog Brain Res.

[105] Walter EJ, Carraretto M (2016). The neurological and cognitive consequences of hyperthermia. Crit Care.

[106] Xiang R, MacLeod IM, Daetwyler HD, de Jong G, O’Connor E, Schrooten C (2021). Genome-wide fine-mapping identifies pleiotropic and functional variants that predict many traits across global cattle populations. Nat Commun 2021.

[107] Weale ME (2010). Quality control for genome-wide association studies. Methods Mol Biol.

[108] Purcell S, Neale B, Todd-Brown K, Thomas L, Ferreira MAR, Bender D (2007). PLINK: a tool set for whole-genome association and population-based linkage analyses. Am J Hum Genet.

[109] Knauer MT, Baitinger DJ (2015). The Sow Body Condition Caliper. Appl Eng Agric.

[110] Sahana G, Cai Z, Sanchez MP, Bouwman AC, Boichard D (2023). Invited review: good practices in genome-wide association studies to identify candidate sequence variants in dairy cattle. J Dairy Sci.

[111] Yang J, Lee SH, Goddard ME, Visscher PM (2011). GCTA: a tool for genome-wide complex trait analysis. Am J Hum Genet.

[112] Yang J, Zaitlen NA, Goddard ME, Visscher PM, Price AL. Advantages and pitfalls in the application of mixed-model association methods. Nature Genetics 2014 46:2. 2014;46:100–6. 10.1038/ng.2876 PMC3989144 24473328

[113] Listgarten J, Lippert C, Kadie CM, Davidson RI, Eskin E, Heckerman D (2012). Improved linear mixed models for genome-wide association studies. Nat Methods 2012.

[114] Lippert C, Listgarten J, Liu Y, Kadie CM, Davidson RI, Heckerman D (2011). FaST linear mixed models for genome-wide association studies. Nat Methods 2011.

[115] van den Berg S, Vandenplas J, van Eeuwijk FA, Lopes MS, Veerkamp RF (2019). Significance testing and genomic inflation factor using high-density genotypes or whole-genome sequence data. J Anim Breed Genet.

[116] Balding DJ (2006). A tutorial on statistical methods for population association studies. Nat Reviews Genet 2006.

[117] Alghamdi J, Padmanabhan S (2014). Fundamentals of complex trait genetics and association studies. Handb Pharmacogenomics Stratified Med.

[118] Li X, Buitenhuis AJ, Lund MS, Li C, Sun D, Zhang Q (2015). Joint genome-wide association study for milk fatty acid traits in Chinese and Danish holstein populations. J Dairy Sci.

[119] Goddard ME, Hayes BJ, Meuwissen THE (2011). Using the genomic relationship matrix to predict the accuracy of genomic selection. J Anim Breed Genet.

[120] Corbin LJ, Liu AYH, Bishop SC, Woolliams JA (2012). Estimation of historical effective population size using linkage disequilibria with marker data. J Anim Breed Genet.

[121] Bolormaa S, Hayes BJ, van der Werf JHJ, Pethick D, Goddard ME, Daetwyler HD (2016). Detailed phenotyping identifies genes with pleiotropic effects on body composition. BMC Genomics.

[122] Fonseca PAS, Suárez-Vega A, Marras G, Cánovas Á (2020). GALLO: an R package for genomic annotation and integration of multiple data sources in livestock for positional candidate loci. Gigascience.

[123] Huang DW, Sherman BT, Lempicki RA (2009). Systematic and integrative analysis of large gene lists using DAVID bioinformatics resources. Nat Protoc.

[124] Sherman BT, Hao M, Qiu J, Jiao X, Baseler MW, Lane HC (2022). DAVID: a web server for functional enrichment analysis and functional annotation of gene lists (2021 update). Nucleic Acids Res.

