# Supplementary material for: Genomic regions, candidate genes, and pleiotropic variants associated with physiological and anatomical indicators of heat stress response in lactating sows
Source: BMC Genomics. 2024 May 13;25:467. doi: 10.1186/s12864-024-10365-4 (PMC11092106; doi:10.1186/s12864-024-10365-4)
Supplement: Supplementary file 1 — Supplementary Material 1 [file 12864_2024_10365_MOESM1_ESM.docx]

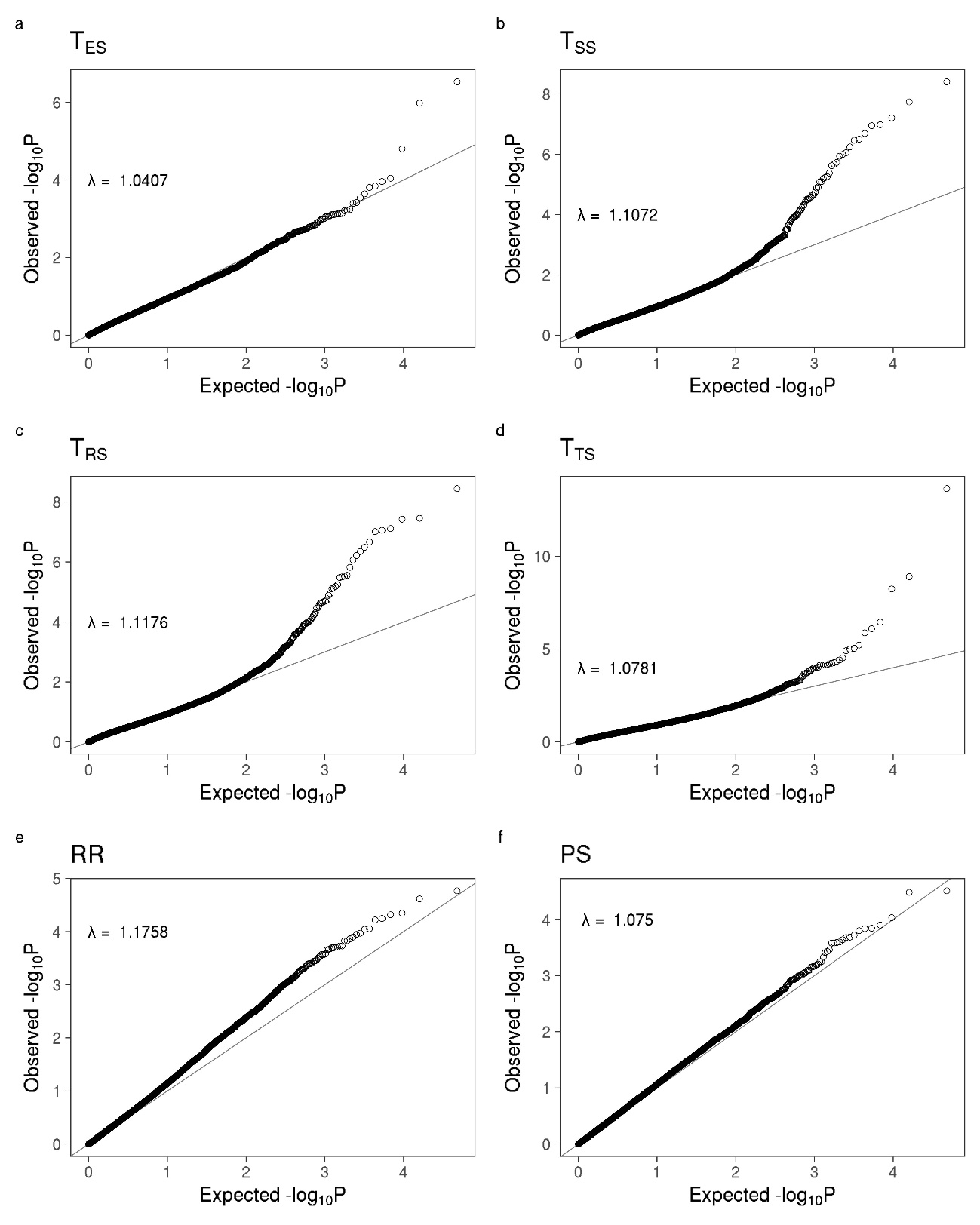


**Figure S1.** Qq-plots for skin temperatures, respiration rate and panting score using medium density genotypes.

-log10(p-values) for ear skin temperature (T_ES_; **a**), shoulder skin temperature (T_SS_; **b**), rump skin temperature (T_RS_; **c**), tail skin temperature (T_TS_; **d**), respiration rate (RR; **e**), and panting score (PS; **f**). Genome-wide significance level shown in a red line.


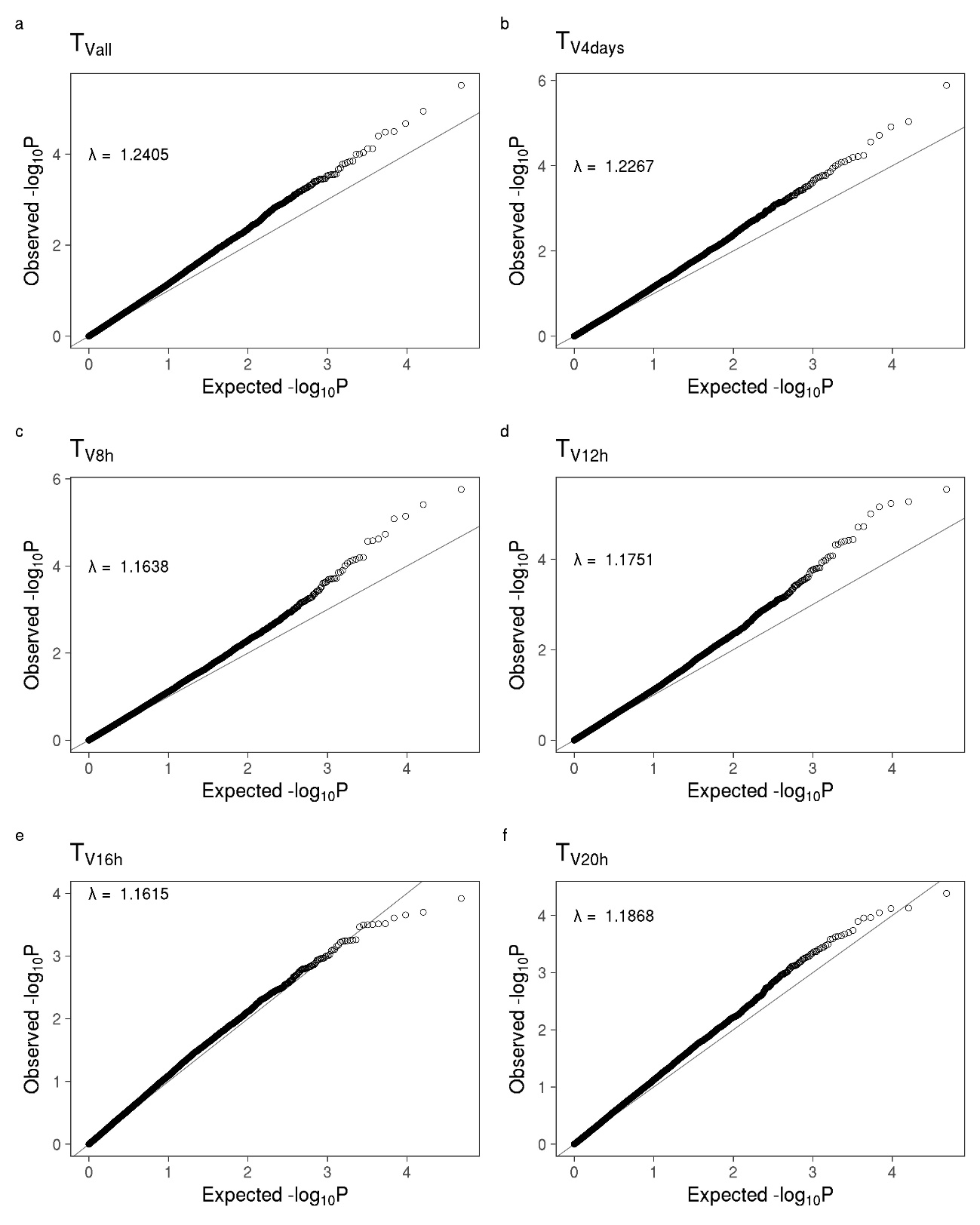


**Figure S2.** Qq-plots of GWAS for vaginal temperatures using medium density genotypes.

-log10(p-values) for all measures (every 10 minutes) of vaginal temperatures for four days (TV_all_; **a**), four-time measures of vaginal temperatures for four days (T_V4days_; **b**), vaginal temperature measured on the first day at 8:00 (T_V8h_; **c**), at 12:00 (T_V12h_; **d**), at 16:00 (T_V16h_; **e**), and at 20:00 (T_V20h_; **f**). Genome-wide significance level shown in a red line.


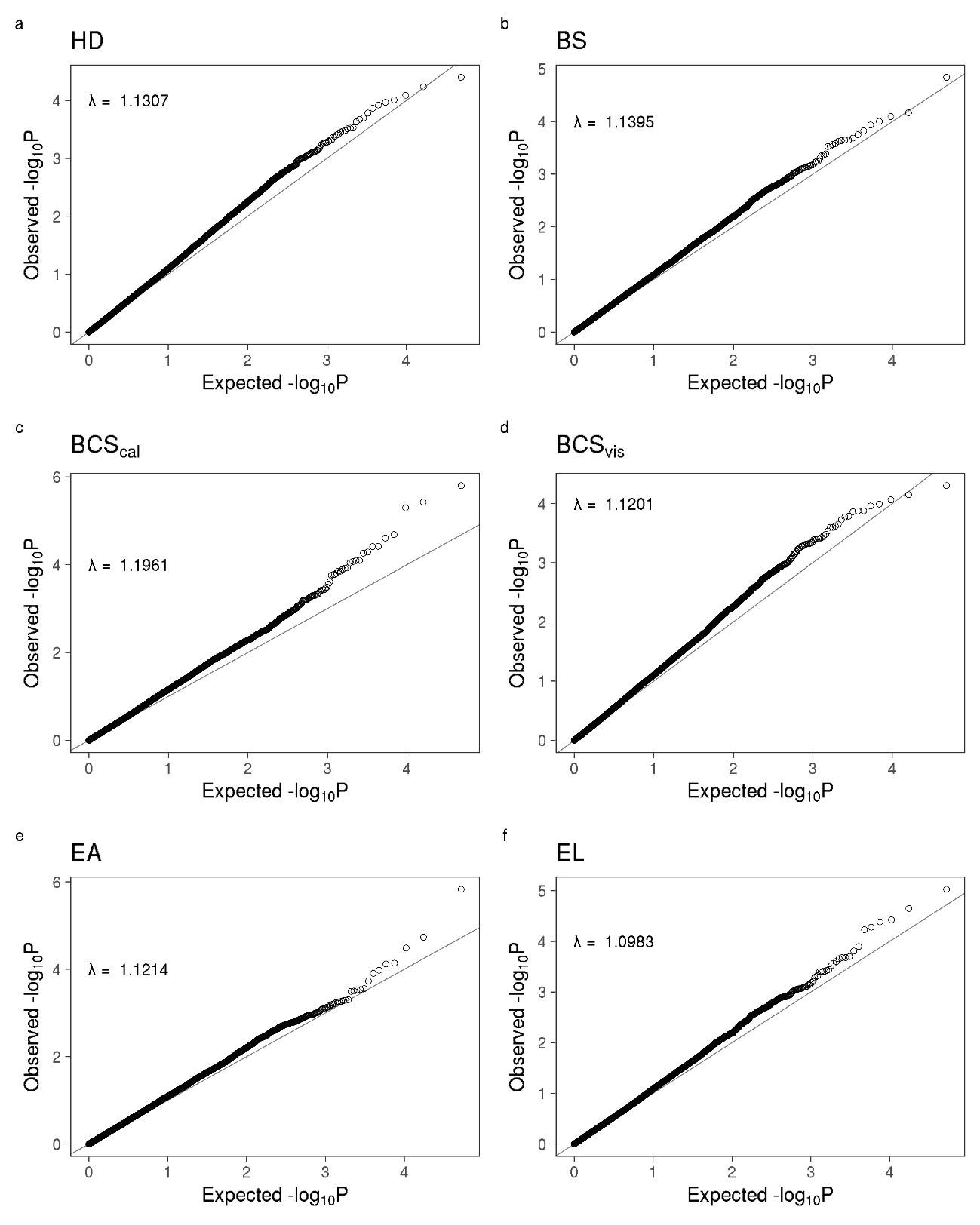


**Figure S3.** Qq-plots of GWAS for anatomical traits using medium density genotypes.

-log10(p-values) for hair density (HD; **a**), body size (BS; **b**), body condition score using a sow caliper (BCS_cal_; **c**) and visual (BCS_vis_; **d**), ear area (EA; **e**), and ear length (EL; **f**). Genome-wide significance level shown in a red line.


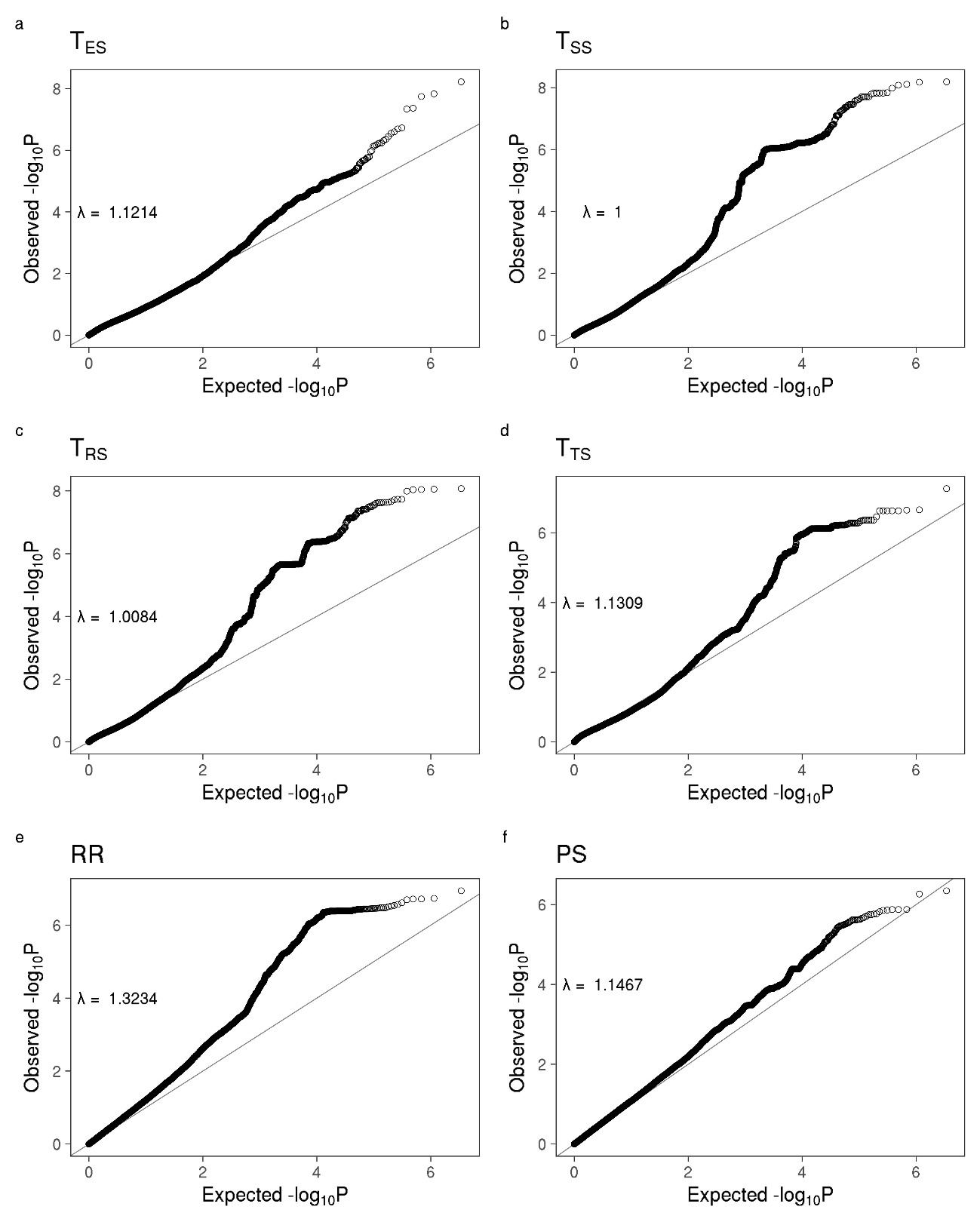


**Figure S4.** Qq-plots for skin temperatures, respiration rate and panting score using whole genome sequence data.

-log10(p-values) for ear skin temperature (T_ES_; **a**), shoulder skin temperature (T_SS_; **b**), rump skin temperature (T_RS_; **c**), tail skin temperature (T_TS_; **d**), respiration rate (RR; **e**), and panting score (PS; **f**). Genome-wide significance level shown in a red line.


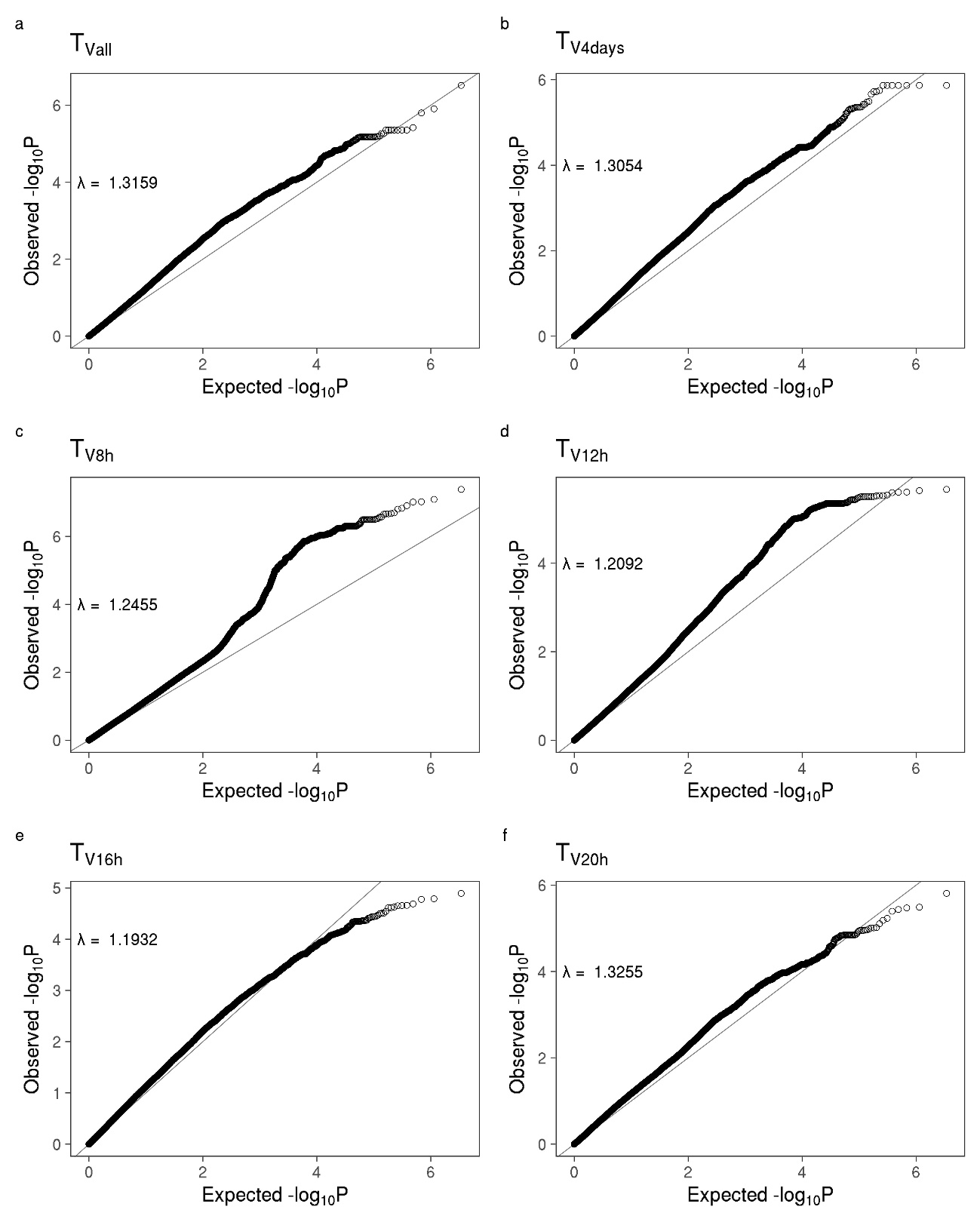


**Figure S5.** Qq-plots of GWAS for vaginal temperatures using whole genome sequence data.

-log10(p-values) for all measures (every 10 minutes) of vaginal temperatures for four days (TV_all_; **a**), four-time measures of vaginal temperatures for four days (T_V4days_; **b**), vaginal temperature measured on the first day at 8:00 (T_V8h_; **c**), at 12:00 (T_V12h_; **d**), at 16:00 (T_V16h_; **e**), and at 20:00 (T_V20h_; **f**). Genome-wide significance level shown in a red line.


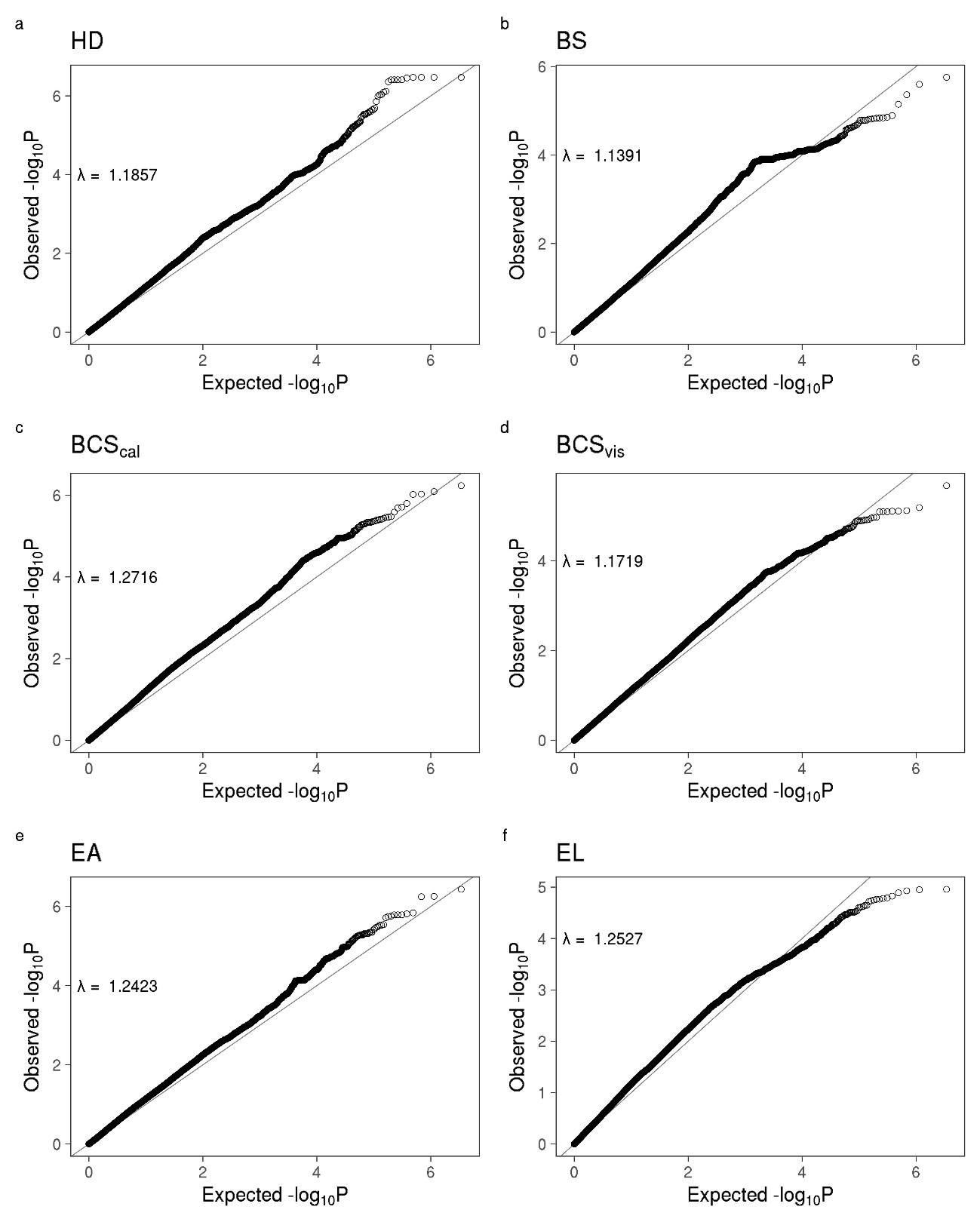


**Figure S6.** Qq-plots of GWAS for anatomical traits using whole genome sequence data.

-log10(p-values) for hair density (HD; **a**), body size (BS; **b**), body condition score using a sow caliper (BCS_cal_; **c**) and visual (BCS_vis_; **d**), ear area (EA; **e**), and ear length (EL; **f**). Genome-wide significance level shown in a red line.


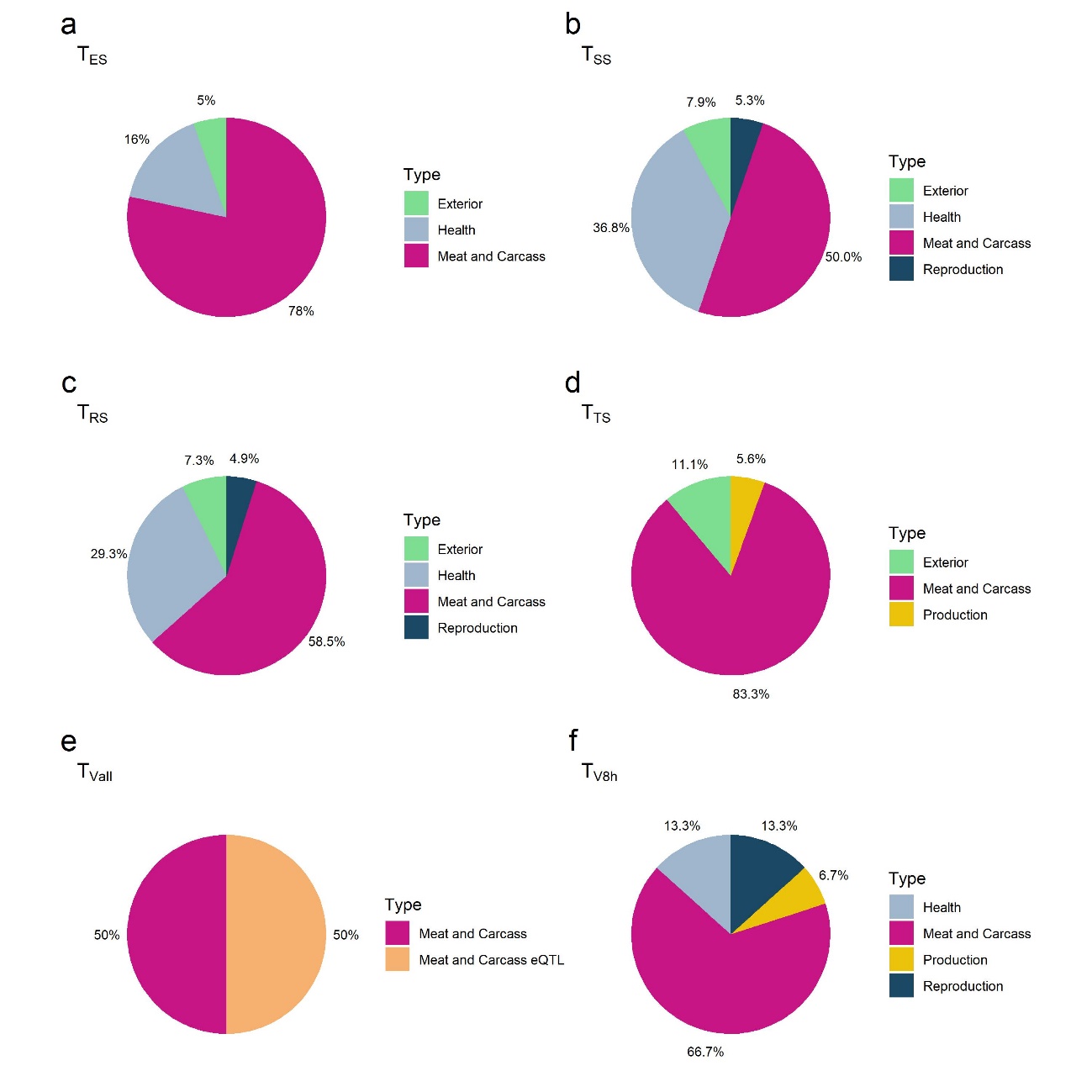


**Figure S7.** Percentage of QTL type for skin temperatures and vaginal temperatures.

Ear skin temperature (T_ES_; **a**), shoulder skin temperature (T_SS_; **b**), rump skin temperature (T_RS_; **c**), tail skin temperature (T_TS_; **d**), all measures (every 10 minutes) of vaginal temperatures for four days (TV_all_; **e**), vaginal temperature measured on the first day at 8:00 (T_V8h_; **f**).


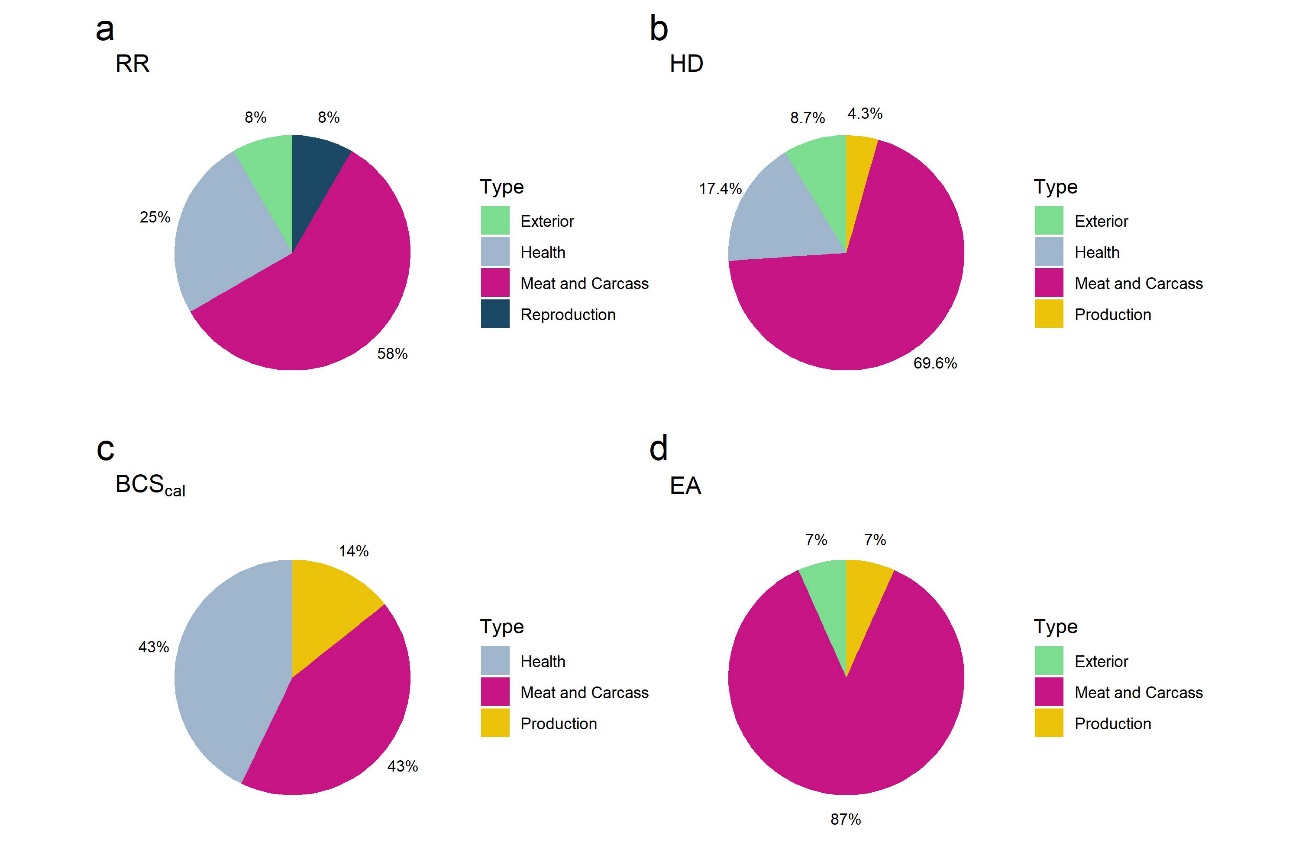


**Figure S8.** Percentage of QTL type for respiration rate and anatomical traits.

Respiration rate (RR, **a**), hair density (HD; **b**), body condition score using a sow caliper (BCS_cal_; **c**) and ear area (EA; **d**).


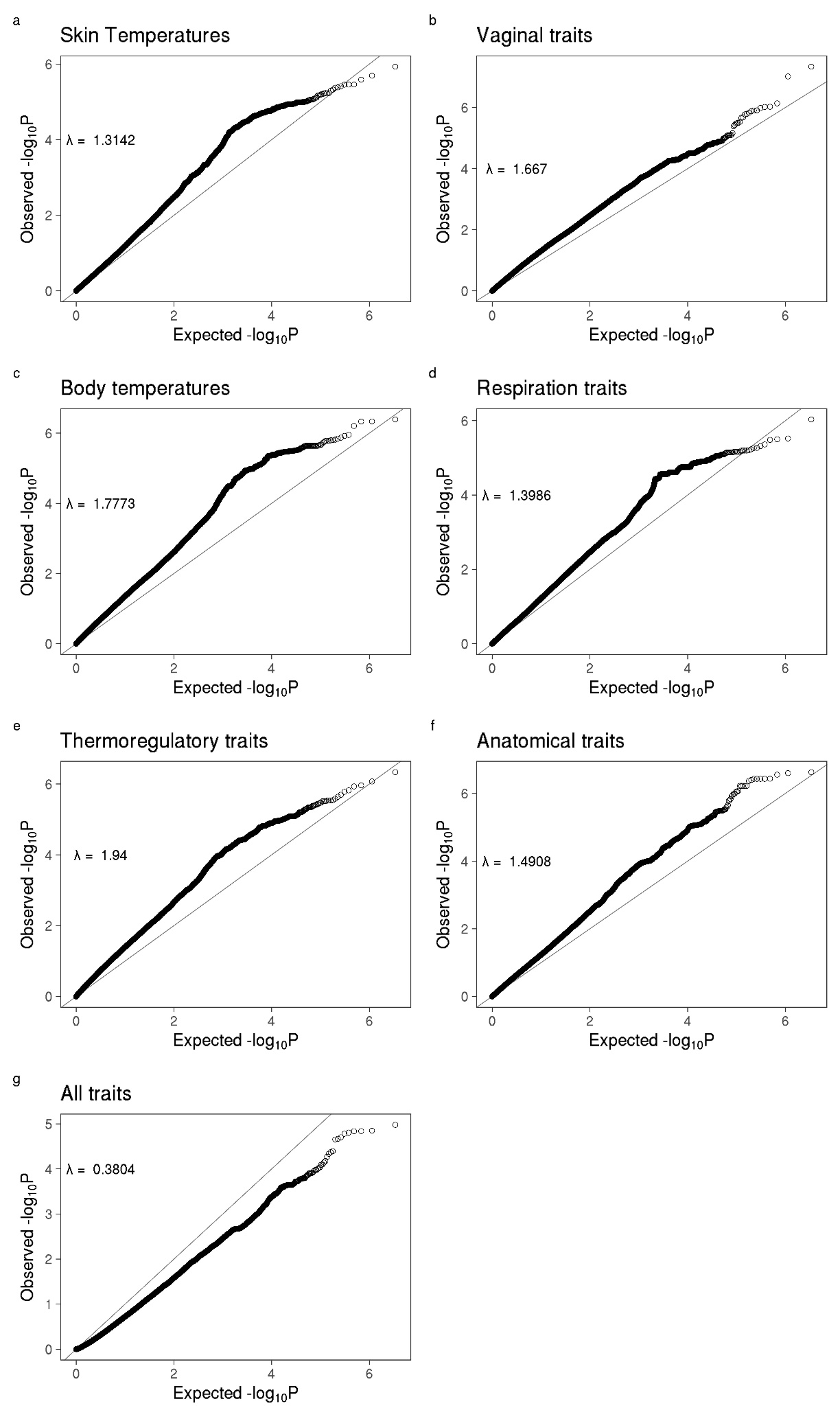


**Figure 9.** Qq-plots for multiple-trait analysis for different categories of traits.

(**a**) The category skin temperatures included the traits ear skin temperature (T_ES_), shoulder skin temperature (T_SS_), rump skin temperature (T_RS_), tail skin temperature (T_TS_). (**b**) The category vaginal temperatures included all measures (every 10 minutes) of vaginal temperatures for four days (TV_all_), four-time measures of vaginal temperatures for four days (T_V4days_), vaginal temperature measured on the first day at 8:00 (T_V8h_), at 12:00 (T_V12h_), at 16:00 (T_V16h_), and at 20:00 (T_V20h_). (**c**) The category body temperature included the traits T_ES_, T_SS_, T_RS_, T_TS_, T_Vall_, T_V4days_, T_V8h_, T_V12h_, T_V16h_, and T_20h_. (**d**) The category respiration traits included respiration rate (RR) and panting score (PS). (**e**) The category thermoregulatory traits included the traits T_ES_, T_SS_, T_RS_, T_TS_, T_Vall_, T_V4days_, T_V8h_, T_V12h_, T_V16h_, T_20h_, RR, and PS. (**f**) The category anatomical traits included the traits hair density (HD), body size (BS), body condition score using a sow caliper (BCS_cal_) and visual (BCS_vis_), ear area (EA), and ear length (EL). (**g**) The category all traits included the traits (T_ES_, T_SS_, T_RS_, T_TS_, T_Vall_, T_V4days_, T_V8h_, T_V12h_, T_V16h_, T_20h_, RR, PS, HD, BS, BCS_cal_, BCS_vis_, EA, and EL).


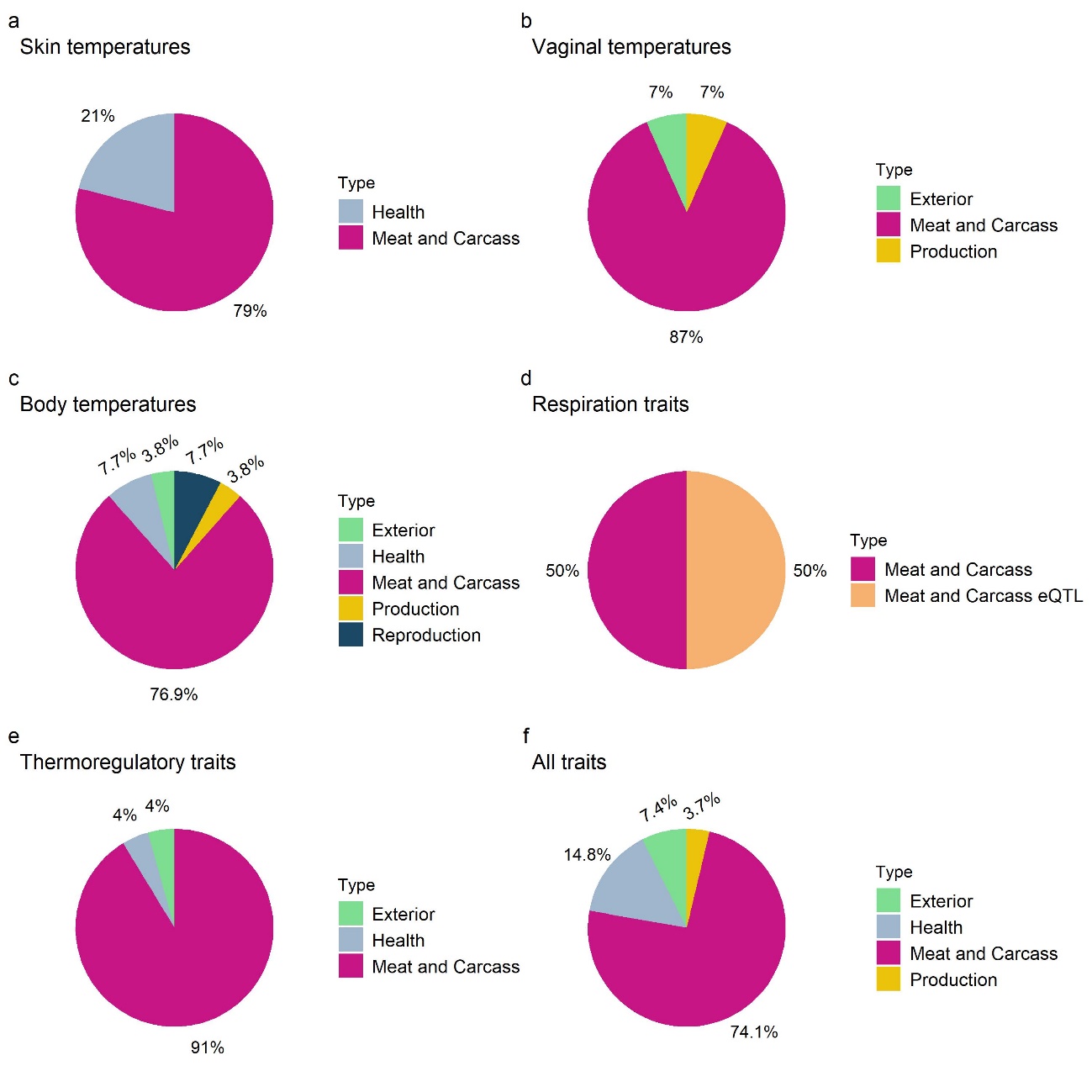


**Figure S10.** Percentage of QTL type for multiple-trait analysis for different categories of traits

(**a**) The category skin temperatures included the traits ear skin temperature (T_ES_), shoulder skin temperature (T_SS_), rump skin temperature (T_RS_), tail skin temperature (T_TS_). (**b**) The category vaginal temperatures included all measures (every 10 minutes) of vaginal temperatures for four days (TV_all_), four-time measures of vaginal temperatures for four days (T_V4days_), vaginal temperature measured on the first day at 8:00 (T_V8h_), at 12:00 (T_V12h_), at 16:00 (T_V16h_), and at 20:00 (T_V20h_). (**c**) The category body temperature included the traits T_ES_, T_SS_, T_RS_, T_TS_, T_Vall_, T_V4days_, T_V8h_, T_V12h_, T_V16h_, and T_20h_. (**d**) The category respiration traits included respiration rate (RR) and panting score (PS). (**e**) The category thermoregulatory traits included the traits T_ES_, T_SS_, T_RS_, T_TS_, T_Vall_, T_V4days_, T_V8h_, T_V12h_, T_V16h_, T_20h_, RR, and PS. (**f**) The category all traits included the traits (T_ES_, T_SS_, T_RS_, T_TS_, T_Vall_, T_V4days_, T_V8h_, T_V12h_, T_V16h_, T_20h_, RR, PS, HD, BS, BCS_cal_, BCS_vis_, EA, and EL).
